# Divergent Reassortment and Transmission Dynamics of Highly Pathogenic Avian Influenza A(H5N8) Virus in Birds of China During 2021

**DOI:** 10.3389/fmicb.2022.913551

**Published:** 2022-06-30

**Authors:** Hejia Ye, Jiahao Zhang, Yunfen Sang, Nan Shan, Weihong Qiu, Wenting Zhong, Junbao Li, Zhaoxia Yuan

**Affiliations:** ^1^College of Animal Science and Technology, Zhongkai University of Agriculture and Engineering, Guangzhou, China; ^2^College of Veterinary Medicine, South China Agricultural University, Guangzhou, China; ^3^Guangzhou South China Biological Medicine, Co., Ltd, Guangzhou, China; ^4^National Avian Influenza Para-Reference Laboratory, Guangzhou, China; ^5^Guangdong Province Key Laboratory of Waterfowl Healthy Breeding, Guangzhou, China; ^6^Nanjing Institute of Environmental Sciences, Ministry of Ecology and Environment of the People’s Republic of China, Nanjing, China; ^7^Key Laboratory of Zoonoses, Ministry of Agriculture and Rural Affairs of the People’s Republic of China, Guangzhou, China

**Keywords:** evolution, H5N8, influenza virus, reassortment, transmission dynamics, BEAST

## Abstract

Highly pathogenic influenza A(H5N8) viruses had caused several outbreaks among wild bird and poultry populations across the globe, and strikingly, caused human infection, posing serious public health concerns. In this study, we conducted influenza surveillance in China during 2021 to monitor the evolution of influenza viruses in poultry. A total of 35 influenza viruses were obtained in chickens, ducks, and geese, of which 30 H5N8 viruses, 3 H5N1 viruses, and 2 H5N6 viruses. Phylogenetic analysis suggested all of H5N1, H5N6, and H5N8 isolates were derived from clade 2.3.4.4b H5N8 viruses during 2020/21 season, and notably, the internal genes of H5N1 and H5N6 viruses shared different genetic heterogeneity with H5N8 viruses and had been reassorted with wild bird-origin H5N1 viruses from Europe. By contrast, almost all H5N8 viruses exhibited only one phylogenic cluster with wild bird-origin H5N8 viruses in China and Korea, indicating that H5N8 viruses in China were more stable. Besides, we found that Korea is the main output geographic location in the spread of these H5N8 viruses to northern and eastern China, and especially, the co-circulation of H5N8 viruses occurred within China, with central China acted as a seeding population during the H5N8 epidemic. The statistical support was strong for viral migration from wild birds to chickens and ducks, indicating that 2.3.4.4b poultry-origin H5N8 viruses during 2020–2021 were originated from wild birds. Our findings provide novel insights into evolution and transmission dynamics of H5 subtype influenza viruses among poultry after novel H5N8 viruses invaded China for nearly one year.

## Introduction

Influenza A viruses not only cause severe economic damage in the poultry but also pose their major threats to human health. Influenza A viruses are classified according to the HA and NA protein spikes into 16 HA and 9 NA subtypes (Webster et al., 1992; Wu et al., 2014; Su et al., 2017), and the H17–18 and N10–11 had been detected in bats (Wu et al., 2014). Aquatic birds, especially migratory birds, are considered the natural reservoirs of highly pathogenic avian H5 subtype viruses (Webster et al., 1992; Venkatesh et al., 2018). The Gs/GD lineage H5N1 viruses evolved rapidly and caused huge poultry industry losses and public health concerns. As of March 2022, a total of 882 human infection with 462 deaths, for a case fatality rate of approximately 52%, posing public health concerns. The rapid evolution of H5 subtype viruses evolved into multiple distinct subclades, among which 2.3.4.4 has become the dominant clade in China (Zhang et al., 2020a). Clade 2.3.4.4 H5Nx influenza viruses were firstly isolated from poultry in 2008 (Bi et al., 2016; Yamaji et al., 2020), and had been co-circulating in wild bird and poultry populations since then. Since 2013, clade 2.3.4.4b H5N8 viruses had been detected in aquatic birds in China, and subsequently, these H5N8 viruses were reported in South Korea (Lee et al., 2014). During 2016/17 wintering season, clade 2.3.4.4b H5N8 viruses were disseminated by migratory birds across the globe to Europe, North America, and Africa, suggestive of the intercontinental spread of influenza viruses (Global Consortium for H5N8 and Related Influenza Virus, 2016; Lee et al., 2017; Yamaji et al., 2020).

Highly pathogenic H5N8 viruses caused numerous outbreaks in central and eastern Europe from January to early June, and subsequently, during the autumn and winter season of 2020, Eurasia experienced a sudden recurrence of H5N8 viruses, and the affected regions extended to Russia, Iraq, Kazakhstan, South Korea, Japan, and China in both poultry and wild birds (Beerens et al., 2021; Lewis et al., 2021; Liang et al., 2021; Shi and Gao, 2021). Strikingly, seven poultry workers infected with novel clade 2.3.4.4b H5N8 viruses were reported in Russia during 2021 (Pyankova et al., 2021), and during the same period, 23 human infection with clade 2.3.4.4b H5N6 viruses had been documented in China.^1^ As of March 2022, a total of 76 human infections with H5N6 viruses had been reported, posing public health threat. Wild waterfowl is regarded as natural reservoirs that contribute to the global spread of avian influenza virus via long-distance migration (Webster et al., 1992; Bi et al., 2016). Recent studies demonstrated that novel 2.3.4.4b H5N8 viruses originating in wild birds frequently invaded multiple provinces of China (Cui et al., 2021; Guo et al., 2021; He et al., 2021; Li et al., 2021a,b,c; Shi and Gao, 2021; Xiong et al., 2021). Frequent contact between wild birds and poultry were regarded as the most probable cause of viral introduction into domestic poultry (Zhang et al., 2020a,^2021^). It is noteworthy that the circulating 2.3.4.4b viruses were antigenically distinct from the clade 2.3.4.4h and 2.3.2.1b vaccine strains in China (Zhang et al., 2021), highlighting the update of H5 subtype vaccine in poultry. The poultry-to-human interface may remain elevated due to possible dissemination of the H5N8 viruses among poultry and wild birds.

To better understand the prevalence, genomic characterization, and ecology of H5 subtype viruses in domestic poultry after H5N8 viruses entered China, here, we conducted influenza surveillance in mainland China, and explored the epidemiology and genomic evolution of H5 subtype viruses among domestic poultry in China during spring to autumn 2021. Our study initially provides novel insights into the ecology, evolution, and transmission dynamics of poultry-origin H5 subtype influenza viruses after novel wild bird-origin 2.3.4.4b H5N8 viruses invaded China, which offers the instructive implication for the prevention of control avian influenza viruses.

## Materials and Methods

### Sample Collection and Virus Isolation

During 2021, we collected cloacal and tracheal swab samples from chickens, ducks, and geese in 12 provinces of China, including Liaoning, Shandong, Henan, Jiangsu, Anhui, Sichuan, Guangxi, Guangdong, Hebei, Fujian, Yunnan, and Hubei provinces. Each sample was placed in 2 ml of the PBS supplemented with penicillin (5,000 U/ml) and streptomycin (5,000 U/ml) and inoculated in the allantoic cavities of 10-day-old specific-pathogen-free (SPF) embryonated chicken eggs at 37°C. The allantoic fluid was collected and tested via HA assay with 1% chicken red blood cells before use.

### RNA Extraction, RT-PCR, and DNA Sequencing

RNA was extracted from the suspension of viral isolates with the RNeasy Mini Kit (QIAGEN) as directed by the manufacturer. Two-step RT-PCR was conducted with universal primers as previously described (Qi et al., 2018; Zhang et al., 2020b). Briefly, RNA was reverse transcribed into cDNA using the M-MLV reverse transcription (TakaRa) with Uni12 primer (5′-AGCAAAAGCAGG-3′) for 1 h at 42°C. Before amplification of the full-length genome sequences, virus isolation from the swab samples was conducted, and the HA and NA genes of positive samples were firstly amplified and identified. Then, the eight full-length genome sequences of H5 subtype viruses (H5N1, H5N6, and H5N8) were amplified using PrimeSTAR Max DNA Polymerase (TakaRa) with frame-specific primers. PCR program was set as follows: initial denaturation at 95°C for 5 min, followed by 30 cycles of 95°C for 30 s, 55°C for 30 s, and 72°C for 2 min, and 72°C for 5 min for final extension. PCR products were purified with a Gel Extraction Kit D2500 (OMEGA) and the gene segments were sequenced by TSINGKE Co., Ltd. (Guangdong, China).

### Phylogenetic Analysis

All eight available genome sequences with the complete coding regions of H5N1, H5N6, and H5N8 viruses were downloaded from GISAID,^2^ after which datasets for the sequences together with our H5N1, H5N6, and H5N8 strains were aligned using MAFFT (version 7.313) program (Katoh et al., 2002). The best fit substitution models for eight gene segments were estimated using Modelfinder (Kalyaanamoorthy et al., 2017) (Supplementary Table 1). Maximum likelihood (ML) phylogenies for the codon alignment of the eight genome sequences were estimated using the best-fit nucleotide substitution model in the IQ-TREE 1.68 software^3^ (Nguyen et al., 2015). Node support was determined by non-parametric bootstrapping with 1,000 replicates, and the phylogenetic tree was visualized in the FigTree (version 1.4.3) program.^4^ The positions of sequences coming from one isolates were traced by line (colored by the different regions, including China, Europe, Russia, South Korea, and Japan, Asian countries except China, Russia, South Korea, and Japan) in eight trees. The R package GGTREE (version 1.16.5) was used to generate the results (Yu et al., 2018).

### Phylodynamic Analysis

Time-measured phylogeny was inferred using Bayesian discreate phylogeographic approach implemented in BEAST package (version 1.8.2) (Drummond et al., 2012). The discrete sampling locations of clade 2.3.4.4b H5 subtype viruses during 2020–2021 including Europe (*n* = 152), Russia (*n* = 44), Korea (*n* = 46), Japan (*n* = 8); Kazakhstan (*n* = 12); Southern and Southwestern China (Guangxi, Guangdong, and Sichuan; *n* = 20); Eastern China (Jiangsu, Anhui, Zhejiang, and Jiangxi; *n* = 8); Central China (Henan and Hubei, *n* = 24); Northern China (Liaoning, Ningxia, Hebei, and Beijing; *n* = 7); Western China (Shanxi and Shaanxi; *n* = 17); Shandong province of China (*n* = 31). The discrete sampling hosts of clade 2.3.4.4b H5 subtype viruses during 2020–2021 including chickens (*n* = 117), ducks (*n* = 80), goose (*n* = 51), and wild birds (*n* = 121). We first performed a regression of root-to-tip genetic distances on the ML tree against exact sampling dates using the TempEst (Rambaut et al., 2016), which showed strong temporal signal. Then, we used the Bayesian Markov chain Monte Carlo (MCMC) method implemented in the BEAST package (version 1.8.2) (Drummond et al., 2012), employing the GTR nucleotide substitution model, an uncorrected lognormal (UCLN) relaxed molecular clock model, and a Bayesian skyline coalescent model (Minin et al., 2008). Besides, a Bayesian stochastic search variable selection (BSSVS) model with asymmetric substitution was conducted. For each independent dataset, multiple runs of MCMC method were combined using LogCombiner (version 1.8.3), utilizing 100,000,000 total steps for each set, with sampling every 10,000 steps. The convergence (i.e., effective sample sizes >200) of relevant parameters was assessed using Tracer (version 1.5).^5^ The posterior distribution of trees obtained from BEAST analysis (with 10% of runs removed as burn-in) was also explored to obtain the MCC tree for HA gene segment. After that, we performed BEAST analysis of HA gene of clade 2.3.4.4b H5N8 viruses from 2020 to 2021 with the same parameters used to estimate relative genetic diversity. All phylogenetic trees were visualized in FigTree (version 1.4.3) program(see text footnote 4) with nodes in ascending order. Subsequently, we used SpreaD3 v0.9.7 (Bielejec et al., 2016) to develop interactive visualizations of the dispersal process through time and to compute a Bayes factors (BFs) test to assess the support for significant individual transitions between distinct geographic locations. SpreaD3 takes a rate matrix file for location states generated under the BEAST analysis using the BSSVS procedure (Bielejec et al., 2016). We used R^6^ and ArcGIS Desktop 10.4 software^7^ to create plots showing results of BF tests.

## Results

### Epidemiology of HPAI H5 Subtype Viruses in China

**FIGURE 1:**
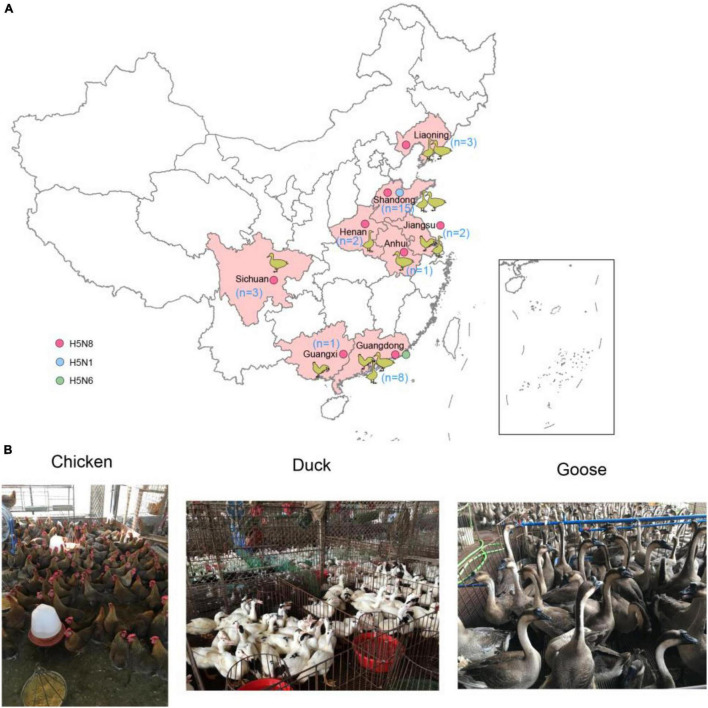
Geographic distribution of H5 subtype influenza viruses isolated from chickens, ducks, and geese in China during January to August 2021 **(A)** and sampling picture in live poultry markets of China **(B)**. The animal cartoon in the map represents the host of H5 subtype viruses in different provinces of China in this study. The blue, green, and red dots represent H5N1, H5N6, and H5N8 viruses in this study. The map in the inset indicates islands in the South China Sea. The map was designed using ArcGIS Desktop 10.4 software (http://www.esri.com/software/arcgis/arcgis-for-desktop/).

We conducted active influenza surveillance in live poultry markets (LPMs) of China during 2021 (Figures 1A,B). The cloacal and tracheal swab samples were collected in chickens, ducks, geese. A total of 35 H5 subtype influenza viruses were detected in eight provinces of China (Table 1). The clade 2.3.4.4b H5N8 viruses were detected in Shandong (*n* = 12), Liaoning (*n* = 3), Jiangsu (*n* = 2), Anhui (*n* = 1), Henan (*n* = 2), Sichuan (*n* = 3), Guangxi (*n* = 1), and Guangdong (*n* = 6) provinces (Figure 1A and Table 1). In addition to the clade 2.3.4.4b H5N8 viruses, the clade 2.3.4.4b H5N1 viruses were also isolated from ducks in Shandong province (*n* = 3), and the clade 2.3.4.4b H5N6 viruses were obtained from chickens in Guangdong province (*n* = 2) (Figure 1A and Table 1). These findings suggested that the H5N1, H5N6, and H5N8 influenza viruses had been co-circulating in poultry in mainland China during 2021. In addition, we also found that approximately 83% of H5 subtype influenza viruses were isolated from aquatic birds, suggesting that aquatic birds were regarded as the reservoir hosts of H5 subtype influenza viruses in China.

**TABLE 1:** Highly pathogenic avian influenza H5 subtype viruses isolated from January 2021 to August 2021.

Isolation name	Location	Collection date	Host	Subtype	Clade
A/Duck/Sichuan/21022-1/2021(H5N8)	Sichuan	Jan-21	Duck	H5N8	2.3.4.4b
A/Goose/Henan/21028/2021(H5N8)	Henan	Jan-21	Goose	H5N8	2.3.4.4b
A/Duck/Sichuan/21044-2/2021(H5N8)	Sichuan	Jan-21	Duck	H5N8	2.3.4.4b
A/Goose/Henan/21056-2/2021(H5N8)	Henan	Jan-21	Goose	H5N8	2.3.4.4b
A/Duck/Guangdong/21057/2021(H5N8)	Guangdong	Jan-21	Duck	H5N8	2.3.4.4b
A/Duck/Sichuan/21022-4/2021(H5N8)	Sichuan	Jan-21	Duck	H5N8	2.3.4.4b
A/Goose/Jiangsu/21153-2/2021(H5N8)	Jiangsu	Feb-21	Goose	H5N8	2.3.4.4b
A/Goose/Shandong/21153-3/2021(H5N8)	Shandong	Feb-21	Goose	H5N8	2.3.4.4b
A/Duck/Shandong/21155-1/2021(H5N8)	Shandong	Feb-21	Duck	H5N8	2.3.4.4b
A/Chicken/Jiangsu/21187/2021(H5N8)	Jiangsu	Feb-21	Chicken	H5N8	2.3.4.4b
A/Chicken/Guangdong/21199-2/2021(H5N8)	Guangdong	Feb-21	Chicken	H5N8	2.3.4.4b
A/Duck/Shandong/21376-1/2021(H5N8)	Shandong	Feb-21	Duck	H5N8	2.3.4.4b
A/Duck/Shandong/21232-5/2021(H5N8)	Shandong	Mar-21	Duck	H5N8	2.3.4.4b
A/Duck/Guangdong/21316/2021(H5N8)	Guangdong	Mar-21	Duck	H5N8	2.3.4.4b
A/Chicken/Liaoning/21346-2/2021(H5N8)	Liaoning	Mar-21	Chicken	H5N8	2.3.4.4b
A/Goose/Shandong/21369-4/2021(H5N8)	Shandong	Mar-21	Goose	H5N8	2.3.4.4b
A/Goose/Shandong/21369-5/2021(H5N8)	Shandong	Mar-21	Goose	H5N8	2.3.4.4b
A/Duck/Shandong/21232-2/2021(H5N1)	Shandong	Mar-21	Duck	H5N1	2.3.4.4b
A/Duck/Shandong/21376-4/2021(H5N1)	Shandong	Mar-21	Duck	H5N1	2.3.4.4b
A/Duck/Shandong/21376-5/2021(H5N8)	Shandong	Mar-21	Duck	H5N8	2.3.4.4b
A/Duck/Shandong/21644-1/2021(H5N8)	Shandong	Mar-21	Duck	H5N8	2.3.4.4b
A/Duck/Shandong/21644-4/2021(H5N8)	Shandong	Apr-21	Duck	H5N8	2.3.4.4b
A/Duck/Shandong/21644-7/2021(H5N8)	Shandong	May-21	Duck	H5N8	2.3.4.4b
A/Goose/Liaoning/21640/2021(H5N8)	Liaoning	Jun-21	Goose	H5N8	2.3.4.4b
A/Goose/Liaoning/21723/2021(H5N8)	Liaoning	Jun-21	Goose	H5N8	2.3.4.4b
A/Duck/Anhui/21931-1/2021(H5N8)	Anhui	Jun-21	Duck	H5N8	2.3.4.4b
A/Duck/Shandong/21931-3/2021(H5N8)	Shandong	Jun-21	Duck	H5N8	2.3.4.4b
A/Duck/Shandong/21931-8/2021(H5N8)	Shandong	Jul-21	Duck	H5N8	2.3.4.4b
A/Chicken/Guangxi/21989-3/2021(H5N8)	Guangxi	Jul-21	Chicken	H5N8	2.3.4.4b
A/Duck/Shandong/21931-6/2021(H5N1)	Shandong	Jul-21	Duck	H5N1	2.3.4.4b
A/Goose/Guangdong/21858/2021(H5N8)	Guangdong	Jul-21	Goose	H5N8	2.3.4.4b
A/Duck/Guangdong/21964/2021(H5N8)	Guangdong	Aug-21	Duck	H5N8	2.3.4.4b
A/Goose/Guangdong/211030-1/2021(H5N8)	Guangdong	Aug-21	Goose	H5N8	2.3.4.4b
A/Chicken/Guangdong/211106-1/2021(H5N6)	Guangdong	Aug-21	Chicken	H5N6	2.3.4.4b
A/Chicken/Guangdong/211106-3/2021(H5N6)	Guangdong	Aug-21	Chicken	H5N6	2.3.4.4b

### Phylogenetic Analysis

**FIGURE 2:**
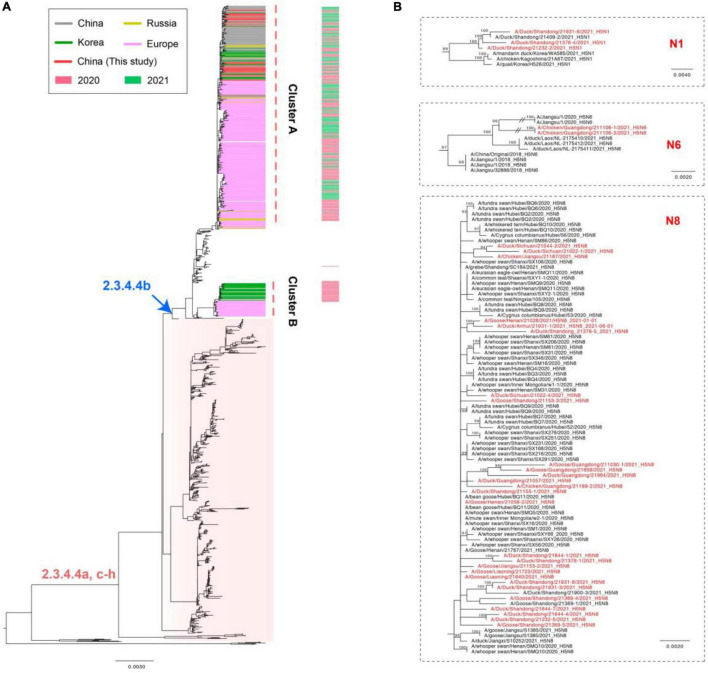
Maximum likelihood (ML) tree of HA **(A)** and NA **(B)** gene sequences of H5 subtype viruses. All of the H5Nx viruses in China, including our 30 H5N8 viruses, 3 H5N1 viruses, 2 H5N6 viruses were used to perform the phylogenic analysis. All branch lengths are scaled according to the numbers of substitutions per site (subs/site). ML phylogenies for the codon alignment of the full gene segments were estimated using the best-fit nucleotide substitution model in the IQ-TREE. Node support was determined by non-parametric bootstrapping with 1,000 replicates. The phylogenetic tree was visualized in the FigTree (version 1.4.3) program.

The whole genomic sequences of 35 avian influenza viruses (AIV), including 30 H5N8 viruses during January to August 2021, 3 H5N1 viruses during March to July 2021, and 2 H5N6 viruses in August 2021. The sequences of all H5 subtype influenza viruses were submitted to the GISAID database^8^ (Supplementary Table 2). To explore genetic relationship of these H5 subtype influenza viruses, we conducted phylogenetic trees of H5 subtype influenza viruses in this study with available gene segments in the GISAID database using IQ-TREE (Nguyen et al., 2015). Phylogenetic analysis demonstrated that HA genes of these H5 subtype influenza viruses clustered together and were derived from clade 2.3.4.4b H5N8 viruses (Figure 2A). It is noteworthy that 2.3.4.4b H5 subtype influenza viruses during 2020/21 wintering seasons were divided into two clusters in our previous study (Zhang et al., 2021), including cluster A and cluster B. In this study, we found that all of our H5 subtype viruses were derived from cluster A (Figure 2A), indicating that these H5 subtype influenza viruses shared a common origin.

In addition, the HA and NA genes of our cluster A H5N8 isolates in China during 2021 and the H5N8 viruses in Europe during 2020 did not cluster together (Figures 2A,B); however, the H5N8 subtype viruses in this study were genetically closely related to the wild-bird-origin H5N8 viruses in China and Korea (Figures 2A,B), which suggested that H5N8 influenza viruses had been co-circulating in China and Korea with frequent wild bird-poultry interface. Besides, the NA genes of 3 H5N1 viruses were genetically closely related to wild bird-origin H5 subtype viruses in Europe (Supplementary Figure 6). The NA genes of 2 H5N6 viruses were derived from local H5N6 viruses in Laos during 2021 and exhibited a long genetic distance to human-infecting H5N6 viruses in Jiangsu during 2018 (Supplementary Figure 7).

**FIGURE 3:**
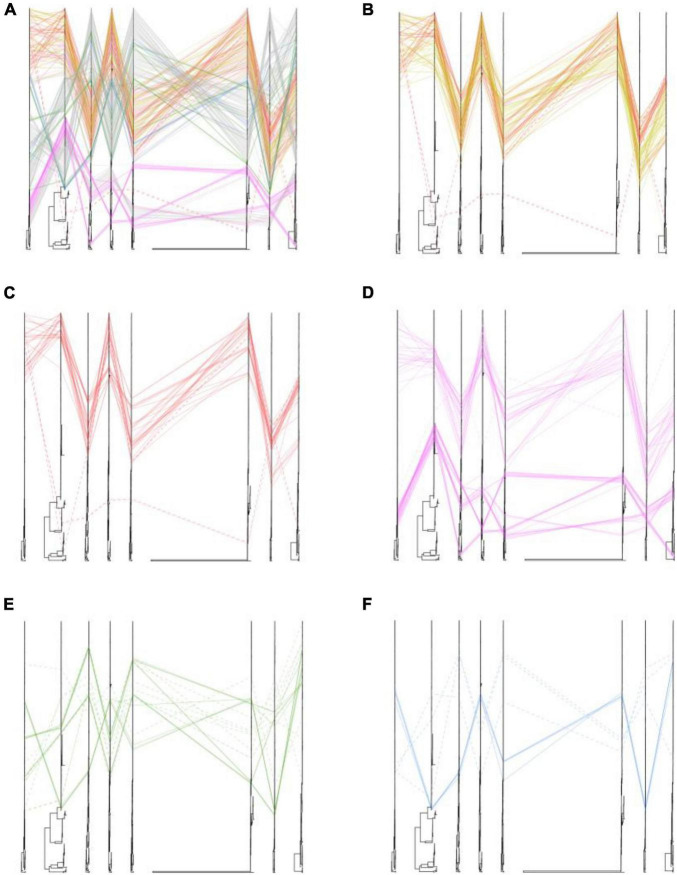
Phylogenetic incongruence analysis of clade 2.3.4.4b H5 subtype influenza viruses during 2020–2021. Maximum likelihood trees for the HA and NA segments as well as all the internal genes, PB2, PB1, PA, NP, MP, and NS from equivalent strains were connected across the trees. Lines are colored according to the different locations. Strains that are clustered together are shown with a solid line, and strains that cannot be classified to any cluster are shown with a dashed line. **(A)** Phylogenetic incongruence of all isolates. **(B)** Phylogenetic incongruence of clade 2.3.4.4b H5 isolates in China during 2020–2021. **(C)** Phylogenetic incongruence of clade 2.3.4.4b H5 isolates in China in this study during 2020–2021. **(D)** Phylogenetic incongruence of clade 2.3.4.4b H5 isolates in Korea during 2020–2021. **(E)** Phylogenetic incongruence of clade 2.3.4.4b H5 isolates in Russia during 2020–2021. **(F)** Phylogenetic incongruence of clade 2.3.4.4b H5 isolates in Bangladesh, Kazakhstan, and Vietnam during 2020–2021. The phylogenetic incongruence was estimated using R package GGTREE (version 1.16.5).

We also placed the phylogenetic incongruence results of H5 subtype viruses with different locations. The positions of sequences coming from one isolates were traced by line in eight trees. In our study, we found that all of the poultry- and wild bird-origin H5N8 viruses in China had only one clear phylogenic cluster; however, different heterogeneity was observed within the H5N1 and H5N6 influenza viruses in our study (Figure 3). Interestingly, the internal genes of H5N6 and H5N8 viruses clustered together and were originated from 2.3.4.4b subclade A H5 subtype viruses during 2020/21 wintering season except with the one strain, A/Chicken/Guangxi/21989-3/2021(H5N8) (hereafter 21989-3). The NS gene of 21989-3 was derived from local H5N6 viruses in Bangladesh and Korea, indicative of minor reassortment between H5N6 and H5N8 viruses (Figure 3 and Supplementary Figures 1–3, 5, 9, 10). All of the H5N1 viruses in this study did not cluster together with H5N6 and H5N8 viruses. Specially, the PB2, PB1, PA, NP, NA, and NS genes were genetically closely related to wild bird-origin H5 subtype viruses from Europe and Korea, with a distant genetic relationship of H5N8 viruses in China (Supplementary Figures 1–3, 5, 10). These findings suggested that the poultry origin clade 2.3.4.4b H5N1 and H5N6 viruses had been reassorted with local H5N6, H5N8 viruses, and wild bird-origin H5 subtype viruses at least twice.

We then compared the differences of genetic heterogeneity of H5N8 viruses in different countries. In contrast to H5N8 viruses in China, the phylogenetic clustering of 2.3.4.4b H5N8 viruses in Europe, Korea, and Russia showed higher heterogeneity (Figure 3). However, the same phylogenetic clustering of 2.3.4.4b cluster A H5N8 viruses were observed in both China and Korea (Figure 3). These findings suggested that these 2.3.4.4b cluster A H5N8 viruses established in poultry and wild birds in China during 2021 stably maintained and had close phylogenetic relationships in neighboring Asian countries.

### Transmission Dynamics Analysis

To further analyze the transmission dynamics of clade 2.3.4.4b H5N8 viruses during 2020–2021, we conducted the dissemination pathways of clade 2.3.4.4b H5N8 viruses during 2020–2021 followed a phylogeographic approach. We grouped H5N8 viruses in China into eleven distinct geographic categories, including Europe (*n* = 152), Russia (*n* = 44), Korea (*n* = 46), Japan (*n* = 8); Kazakhstan (*n* = 12); Southern and Southwestern China (*n* = 20); Eastern China (*n* = 8); Central China (*n* = 24); Northern China (*n* = 7); Western China (*n* = 17); Shandong province of China (*n* = 31). Transmission routes with BF values >3 and posterior probability >0.5 were selected for analysis.

**FIGURE 4:**
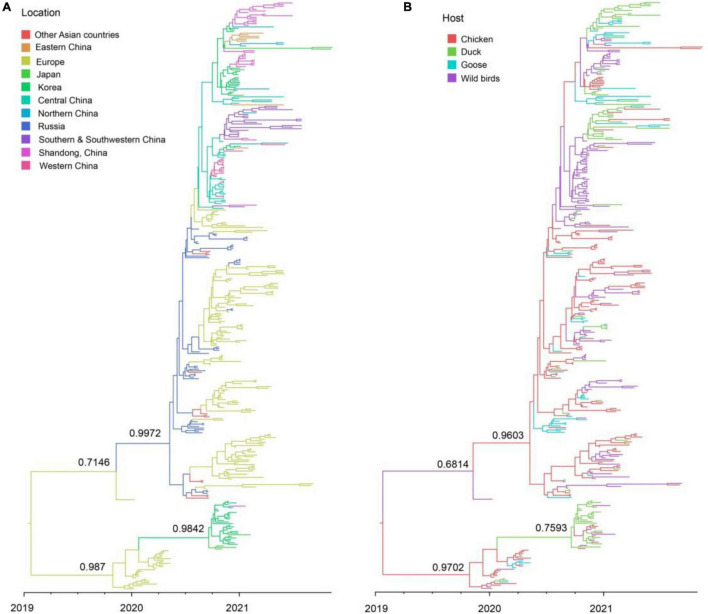
Maximum clade credibility (MCC) time-scaled phylogenetic tree of HA sequences of clade 2.3.4.4b H5 subtype influenza viruses during 2020–2021 colored by geographic location **(A)**, and host species **(B)**. The branches are colored according to the most probable ancestral geographic location, and host type. The phylogenetic tree was visualized in the FigTree (version 1.4.3) program.

The clade 2.3.4.4b H5N8 viruses during 2020–2021 were clustered into two clusters, A and B. Cluster A H5N8 viruses had been dominated and widely spread in multiple countries (Figure 4). Cluster B H5N8 viruses were mainly originated from Europe and Korea, which the time of the most recent common ancestors (tMRCA) was much earlier than that of the cluster A H5N8 viruses (Figure 4). In the Bayesian maximum clade credibility tree, we found that Europe was the geographic origin of cluster A H5N8 viruses during 2020–2021, with a posterior probability of 0.7146, and notably, all of the cluster A H5N8 viruses were originated from wild birds, with a posterior probability of 0.6814 (Figure 4), suggestive of wild birds origin.

**FIGURE 5:**
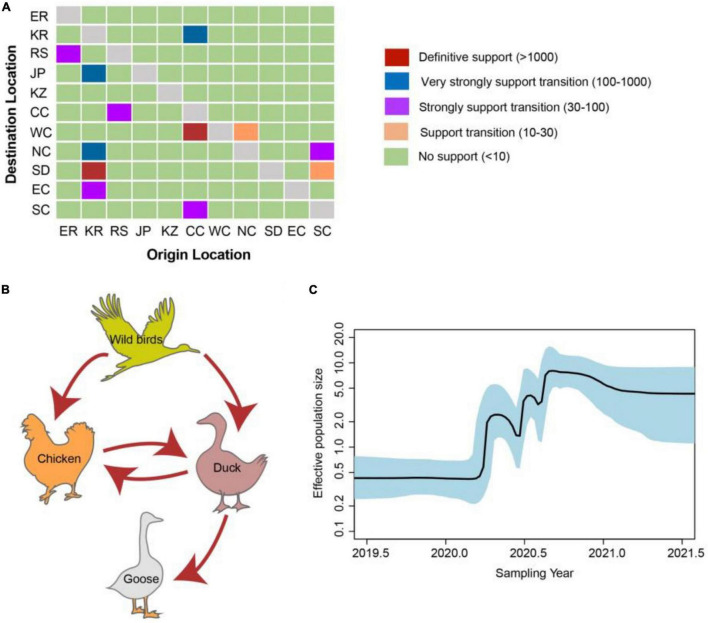
Evolutionary dynamics of clade 2.3.4.4b H5 subtype influenza viruses during 2020–2021 and level of Bayes factor (BF) support for each transmission routes. **(A)** The transmission of clade 2.3.4.4b H5 subtype influenza viruses during 2020–2021. The left and right panels display the level of Bayes Factor support for each of the transmission routes considered for H5 subtype viruses. The x-axis represents the origin location, and the y-axis represents the destination. ER, Europe; KR, Korea; RS, Russia; JP, Japan; KZ, Kazakhstan; CC, Central China; WC, Western China; NC, Northern China; SD, Shandong province of China; EC, Eastern China; SC, Southern & Southwestern China. **(B)** Host transition of clade 2.3.4.4b H5 subtype influenza viruses during 2020–2021. Analyzing transition routes with BF values exceeding 3 were selected for analysis. The red arrowheads represent BF exceeding 100. **(C)** Bayesian Skyline plot of HA gene of clade 2.3.4.4b H5 subtype influenza viruses during 2020–2021. A Bayesian Skyline analysis of HA gene of clade 2.3.4.4b H5 subtype influenza viruses during 2020–2021 to display changes in the effective population size over time. The solid blue line indicates the median value, and the shaded blue area represents the 95% highest posterior density of genetic diversity estimates. The Bayesian Skyline plot was estimated using R package (version 1.16.5).

**FIGURE 6:**
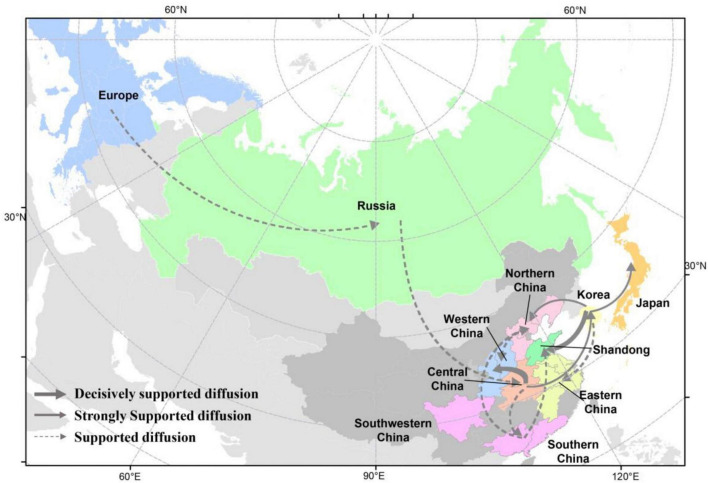
Spatial diffusion of the HA gene segment of clade 2.3.4.4b H5 subtype viruses during 2020–2021. The red dots indicate the sampling sites of poultry in China, and the blue dots indicate the sampling sites of wild birds in China. Curves show the among-province or country virus lineage transitions statistically supported with Bayes factor >3 for H5 subtype influenza viruses. The strongly bold gray arrow indicates decisively supported diffusions (BF < 100,000); bold gray arrows, strongly supported diffusions (100 ≤ BF < 10,000); and solid gray arrows, supported diffusions (10 ≤ BF < 100). The map was designed using R and ArcGIS Desktop 10.4 software (http://www.esri.com/software/arcgis/arcgis-for-desktop/).

There were 12 significant transmission routes of clade 2.3.4.4b H5N8 viruses during 2020–2021 (Table 2). Our findings suggested that H5N8 viruses during 2020–2021 in Russia were originated from Europe (BF = 73); however, the migration rate from Europe to Russia was relatively low (Table 2), indicative of the long-distance migration in wild birds carrying H5N8 viruses. In addition, Korea play an important role in the dissemination of H5N8 viruses. There were five dissemination pathways in spatial diffusion of Korea, with four originating from Korea to Northern China (BF = 105), Shandong (BF = 9970), Eastern China (BF = 51), and Japan (BF = 320), and one from Central China to Korea (BF = 288) (Figures 5A, 6 and Table 2). This suggested that Korea is the main output geographic location in the spread of H5N8 viruses to neighboring provinces of China. Besides, the co-circulation of H5N8 viruses during 2021 occurred within different locations of China and Korea, including from Central China to Southern and Southwestern China (BF = 96), Western China (BF = 99784), and Korea (BF = 288) (Figures 5A, 6 and Table 2), indicating that Central China acted as a seeding population during the H5N8 epidemic. However, the transmission routes from Southern to Northern China were also observed in our study (Figure 6). These all suggested that H5N8 viruses in China during 2021 had been well-established and co-circulating via trans-provincial dissemination probably due to live poultry trade or wild bird migration.

**TABLE 2:** Statistically supported Bayes factors and migration rates of clade 2.3.4.4b H5N8 viruses during 2020–2021 estimated from HA gene sequences.

From	To	Bayes factor	Posterior probability	Migration rates
Europe	Russia	73	0.89	0.58
Korea	Northern China	105	0.92	1.24
Korea	Shandong	9970	1.00	1.70
Central China	Southern and Southwestern China	96	0.91	0.93
Central China	Western China	99784	1.00	1.52
Northern China	Western China	17	0.65	0.81
Southern and Southwestern China	Shandong	15	0.62	0.97
Korea	Eastern China	51	0.85	1.24
Korea	Japan	320	0.97	1.58
Central China	Korea	288	0.97	1.35
Russia	Central China	31	0.77	0.82
Southern & Southwestern China	Northern China	58	0.86	0.63

To estimate the transmission of clade 2.3.4.4b H5N8 viruses among different hosts during 2020–2021, we applied a BSSVS procedure to transmission data for chicken, duck, goose, and wild birds. Statistical support was strong for viral migration from wild birds to chickens (BF = 369) and ducks (BF = 355) (Figure 5B and Table 3), which strongly suggested that 2.3.4.4b poultry-origin H5N8 viruses during 2020–2021 were originated from wild birds. Besides, we also found that the co-circulation of H5N8 viruses occurred in chicken and duck origin (Figure 5B). The cross-species transmission from duck to wild birds (BF = 10), from goose to wild birds (BF = 13) were also observed (Table 3), although the BFs of these routes were relatively low. No transmission routes from chicken to wild birds were observed in this study. These suggested that aquatic birds, especially wild birds, play an important role in the transmission of H5N8 viruses.

**TABLE 3:** Statistically supported host transition of clade 2.3.4.4b H5N8 viruses during 2020–2021 estimated from HA gene sequences.

From	To	Bayes factor	Posterior probability	Migration rates
Chicken	Duck	47,181	1.00	1.16
Duck	Goose	104	0.98	0.73
Duck	Wild birds	10	0.82	0.78
Goose	Wild birds	13	0.85	0.61
Duck	Chicken	5,896	1.00	1.18
Goose	Chicken	4	0.64	0.59
Wild bird	Chicken	369	1.00	0.94
Goose	Duck	8	0.78	0.58
Wild bird	Duck	355	0.99	0.99

In addition, we conducted the population dynamics of clade 2.3.4.4b H5N8 viruses during 2020–2021 among wild bird and poultry populations. Our finding suggested that the effective population size of H5N8 viruses increased sharply from spring 2020 and then stably maintained, followed by several fluctuations and reached a peak during mid-2020 (Figure 5C). The effective population size of H5N8 viruses subsequently stably maintained (Figure 5C), indicating that the 2.3.4.4b cluster A H5N8 viruses during 2021 were more stable than that of the previous wave.

### Molecular Characterization

Molecular analysis of HA cleavage sites indicated these H5N1, H5N6, and H5N8 isolates contains multiple basic amino acids, REKRRKR/GLF, which is the characteristic of highly pathogenic avian influenza (HPAI) viruses (Chen et al., 1998). The receptor binding sites 222 and 224 (H5N1 numbering) were Q and G (Chutinimitkul et al., 2010), respectively. However, several substitutions of S133A, S155N, T156A, and T188I, and K218Q in HA protein were observed in these isolates (Yang et al., 2007; Chutinimitkul et al., 2010; Wang et al., 2010; Li et al., 2021c) (Supplementary Table 3), suggesting that these isolates may prefer binding to human-like receptors. We did not find A588V, E627K and D701N in PB2 protein in our isolates (Supplementary Table 4), indicating that these H5 subtype viruses had not adapted to human (Xiao et al., 2016; Qi et al., 2018). Furthermore, D3V and D622G in PB1 protein, N383D in PA protein, M105V and A184K in NP protein, and N30D, I42M, and T215A in M1 protein were observed in these H5 subtype isolates (Supplementary Tables 5–9), which suggested that these H5 subtype influenza viruses increased the virulence in mice (Fan et al., 2009; Wasilenko et al., 2009; Tada et al., 2011; Nao et al., 2015; Song et al., 2015).

## Discussion

The re-emergence of novel clade 2.3.4.4b H5N8 viruses during 2020–2021 in wild birds and poultry posed a serious threat to not only poultry industry but also human health (Li et al., 2021a,b,c; Pyankova et al., 2021; Zhang et al., 2021). These novel H5N8 viruses repeated invaded China via wild bird migration, causing several outbreaks among migratory birds in China during 2020–2021 wintering season. It is become of increasingly apparent that whether the introduction of these novel H5N8 viruses shape the ecology of H5 subtype viruses in domestic poultry in China, posing public health concerns. Therefore, the comprehensive influenza surveillance among poultry in China is urgently needed.

In our present study, we isolated 35 influenza A H5 subtype viruses from domestic poultry in Southern China (Guangxi and Guangdong), Southwestern China (Sichuan), Northern China (Hebei, Shandong, Anhui, and Liaoning), Western China (Shanxi and Shaanxi), and Eastern China (Anhui, Zhejiang, Jiangsu, and Jiangxi), including 30 H5N8 viruses, 3 H5N1 viruses, and 2 H5N6 viruses. Our study demonstrated that all of these H5 subtype viruses were derived from clade 2.3.4.4b H5N8 viruses during 2020–2021, suggesting that these H5 subtype viruses shared a common origin. In previous study, H5N6 had been replaced H5N1 as the dominant subtype in southern China (Bi et al., 2016); however, the number of subclade 2.3.4.4b H5 subtype viruses increased in poultry population in China during 2021 after H5N8 viruses invaded China. In addition, 83% of H5 subtype influenza viruses were isolated from aquatic birds, most likely due to large population of waterfowl in China (Huang et al., 2012; Zou et al., 2016), which are regarded to be a dominant reservoir of H5 subtype influenza viruses.

In our previous study, we found that clade 2.3.4.4b H5 subtype viruses during 2020–2021 were divided into two clusters, cluster A and cluster B (Zhang et al., 2021). In this study, all of the H5N1, H5N6, and H5N8 viruses were originated from cluster A, which suggested that cluster A in China were stably maintained in poultry for a period. A large quantity of cluster A H5N8 viruses were isolated from wild birds in China (Cui et al., 2021; He et al., 2021), revealing that frequent interface between wild birds and poultry accelerated the co-circulation of cluster A H5N8 viruses. Interestingly, we observed that phylogenetic clustering of 2.3.4.4b H5N8 viruses in Europe, Korea, and Russia during 2020–2021 showed higher heterogeneity. However, the same phylogenetic clustering was observed in almost all our H5N8 isolates, suggested that few reassortment events occurred in H5N8 viruses in poultry of China. It is noteworthy that the internal genes of H5N1 virus were genetically closely related to wild bird-origin H5N1 viruses in Eurasia. The migratory birds and domestic poultry generally share common feeding sites around wetlands (Cappelle et al., 2014), and notably, AIVs can be transmitted between wild birds and domestic poultry. Previous study demonstrated that the direct and speed of the spread of AIV is determined by interdependent factors, including wild bird migration, the live poultry trade, and human population density (Su et al., 2015, 2017; Liu et al., 2018; Yang et al., 2020). The genomes of H5N1 viruses isolated from Shandong province of China were genetically closely related to wild bird-origin H5N1 viruses in Eurasia, indicating that H5N1 viruses have expanded along distinct geographical pathways, likely via long-distance migratory bird dispersal onto several continents, and further evolved by reassorting with local H5 subtype viruses, since Shandong province is located with East Asian-Australian migratory flyways. The co-circulation of influenza viruses in wild birds and domestic poultry, and mammals accelerate the transmission and evolution of influenza viruses, which shapes the ecology of AIV. Thus, it has been inferred that migratory birds play an important role in the dissemination of H5 subtype viruses.

The analysis of HA gene segments using phylogeographic approach suggested that cluster A H5N8 viruses were originated from wild bird-origin H5N8 viruses from Europe. However, we cannot exclude the possibility that H5N8 viruses were originated from outside Europe due to the limited sequence data available. In this study, Korea is the main output geographic location in the spread of these H5N8 viruses to northern and eastern China that were adjacent to Korea, suggested that clade 2.3.4.4b H5N8 viruses in Korea during 2020–2021 has expanded in distinct geographic pathways, likely via short-distance migratory bird dispersal into multiple breeding sites of China (Bourouiba et al., 2010; Hill et al., 2015; Fusaro et al., 2019). Based on our results, we strongly showed the evidence that poultry-origin H5N8 viruses were originated from wild birds. Multiple independent migration pathways of wild birds flowed frequently into China, after which the virus subsequently circulated in various domestic poultry, and the co-circulation of H5N8 viruses between chickens and ducks were also observed, indicating that the transmission of wild birds and poultry shapes the ecology of H5N8 viruses. In addition, Central China acted as a seeding population during the H5N8 epidemic. Central China was linked with three locations, including Southern, Southwestern China, and Western China, which suggested that these H5N8 viruses endemic in Central China might have jointly resulted from the gene flow through circular poultry movements or wild bird migration (Hill et al., 2015; Xu et al., 2016; Li et al., 2018; Zhang et al., 2018, 2020a; Yang et al., 2020). In the case of Hubei province, the site of East Lake, as well as Yellow River Wetland in Sanmenxia of Henan province, with its excellent ecology, vast wetlands, and luxuriant aquatic plants, has become a world-famous migratory bird migration and wintering hub (Li et al., 2021a; Xiong et al., 2021). Because the production cycle of domestic ducks is synchronized with the migration of thousands of migratory birds in the area, H5N8 viruses can frequently transmitted between waterfowl, which raises the risk of the exposure between wild birds and domestic poultry.

Previous study had demonstrated that H5N6 had replaced H5N1 as the dominant subtype in southern China (Bi et al., 2016). However, during 2021, a large amount of clade 2.3.4.4b H5N8 viruses from wild birds and poultry were isolated. The numbers of N8 are increasing. The HA-NA matching fitness bears an important influence on the replication of tissue culture, embryonated chicken eggs, transmission in chickens and ferrets, and pathogenicity in mice (Mitnaul et al., 2000; Baigent and McCauley, 2001; Yen et al., 2011; Yu et al., 2017). Previous study had demonstrated that H5-N6 and H5-N8 matching fitness were greater than H5-N1 matching fitness (Yu et al., 2017), indicating that current circulating H5N8 viruses were more likely adapted to birds. There remains an important question to answer regarding whether these H5N8 viruses could become the dominant subtype in domestic poultry in China. Our previous study showed that these 2.3.4.4b H5N8 viruses were antigenically distinct from the commercial vaccine strains in China (Zhang et al., 2021). In China, vaccination of chickens can be a major strategy for controlling the spread of AIVs in poultry. Since an influenza H5/H7 bivalent vaccine for poultry was introduced in September 2017, the prevalence of the H7N9 viruses in birds and humans decreased sharply (Shi et al., 2018; Wei and Cui, 2018; Zeng et al., 2018; Zhang et al., 2020b), highlighting that clade 2.3.4.4b H5N8 viruses continue to circulate in poultry due to the unmatched H5 vaccine. The HA gene of the vaccine strains used in China since 2004 has been updated several times to ensure an antigenic match between the prevalent H5 subtype strains and vaccine strains (Zhang et al., 2020a). Therefore, we have timely updated the trivalent H5/H7 vaccines, which was approved application in poultry by Ministry of Agricultural and Rural Affairs of the People’s Republic of China in January 27, 2022.^9^

The global spread of novel H5N8 viruses highlight the increasing risk of causing human infection. On December 2020, one zoonotic event of seven poultry workers infected with novel H5N8 viruses were reported in Russia (Pyankova et al., 2021). This is the first report of the human infected with H5N8 AIVs, which signs an alarming threat to public health. Although no well-known mammalian adaption mutations including A588V, E627K and D701N in PB2 protein were observed in our H5 isolates, several substitutions of S133A, S134N, and T156A in HA protein were found in these H5 subtype viruses, suggesting that these novel viruses increased the affinity to bind to α2,6 human-type receptors (Li et al., 2021c). However, recent studies demonstrated that the risk for human-to-human transmission of clade 2.3.4.4b H5N8 viruses seems to be low (Bui et al., 2021), suggesting that these novel H5N8 viruses could not cause pandemic among humans. Recent study demonstrated that compared to individuals exposed to cases of H7N9 viruses, a significantly higher probability of infection was observed in the individuals exposed to H5N1 cases (Chen et al., 2022), indicating that H5N1 was more transmissible in human populations. However, other study showed that human risk following exposure to the European H5N6 viruses remains low. However, of particularly note, during 2021, a sharp increase of human cases infected with clade 2.3.4.4b H5N6 viruses were reported in China (Xiao et al., 2021), which indicated that these novel H5N8 viruses had been continuously reassorted with other subtype viruses, highlighting the increasing threat of 2.3.4.4b H5 viruses posed to humans. In December 2021, an outbreak in poultry in Newfoundland, Canada, marked the second arrival of Eurasian H5 subtype influenza viruses in North America, thereby risking human infection globally.

This study has several limitations. Firstly, the study focus on the epidemiology, evolution, and transmission of H5 subtype AIV from domestic poultry of China; however, the ecology, migration, populations, and species of wild migratory birds in China is also important to clarify. Secondly, although the study had make a conclusion that poultry-origin clade 2.3.4.4b H5 subtype viruses were originated from wild birds; however, there is no concrete evidence even the mechanism of how wild birds aid in mutation of H5 subtype viruses, which requires further investigation.

Taken together, our findings offer novel insights into the genomic evolution and transmission dynamics of H5 subtype influenza viruses among domestic poultry in China during 2021. These 2.3.4.4b H5 subtype viruses had been widespread in poultry in multiple provinces of China, and notably, the co-circulation of H5 subtype viruses were observed in China trans-provincially and with intercontinental spread, especially in central China. The broader prevalence and rapid spread of H5 subtype viruses pose considerable threat to public health. As such, the comprehensive influenza surveillance and the update of vaccines are strongly recommended to prevent potential influenza pandemics in the future.

## Data Availability Statement

The original contributions presented in this study are included in the article/Supplementary Material, further inquiries can be directed to the corresponding author.

## Author Contributions

HY, JZ, and ZY planned and conceptualized the laboratory work and wrote the manuscript draft. HY, YS, WQ, JL, and WZ conducted the virus isolation from samples and sequenced the genomes. JZ and YS performed the bioinformatics analysis and data interpretation. ZY provided the project funding. All authors contributed to the article and approved the submitted version.

## Conflict of Interest

HY, WQ, JL, and WZ were employed by Guangzhou South China Biological Medicine, Co., Ltd, Guangzhou, China. The remaining authors declare that the research was conducted in the absence of any commercial or financial relationships that could be construed as a potential conflict of interest.

## Publisher’s Note

All claims expressed in this article are solely those of the authors and do not necessarily represent those of their affiliated organizations, or those of the publisher, the editors and the reviewers. Any product that may be evaluated in this article, or claim that may be made by its manufacturer, is not guaranteed or endorsed by the publisher.

## Footnotes

1 https://www.who.int/publications/m/item/assessment-of-risk-associated-with-influenza-a(h5n6)-virus

2
http://www.gisaid.org/

3
http://www.iqtree.org/release/v1.6.0

4
http://tree.bio.ed.ac.uk/software/figtree/

5
http://beast.bio.ed.ac.uk/

6
https://www.r-project.org

7
http://www.esri.com/software/arcgis/arcgis-for-desktop/

8
https://platform.epicov.org/epi3/frontend#ec4ba

9
http://www.moa.gov.cn/govpublic/xmsyj/202201/t20220129_6387920.htm

## Supplementary Material

Supplementary Figure 1 Phylogenic tree of H5 subtype influenza viruses using PB2 gene sequences with available the other influenza viruses. The PB2 genes of H5 subtype viruses collected during 2021 in China together with the other influenza viruses were analyzed. The isolates with red color denote the H5N1, H5N6, and H5N8 viruses used in this study. The scale bar represents the number of nucleotide substitutions per site (subs/site).

Supplementary Figure 2 Phylogenic tree of H5 subtype influenza viruses using PB1 gene sequences with available the other influenza viruses. The PB1 genes of H5 subtype viruses collected during 2021 in China together with the other influenza viruses were analyzed. The isolates with red color denote the H5N1, H5N6, and H5N8 viruses used in this study. The scale bar represents the number of nucleotide substitutions per site (subs/site).

Supplementary Figure 3 Phylogenic tree of H5 subtype influenza viruses using PA gene sequences with available the other influenza viruses. The PA genes of H5 subtype viruses collected during 2021 in China together with the other influenza viruses were analyzed. The isolates with red color denote the H5N1, H5N6, and H5N8 viruses used in this study. The scale bar represents the number of nucleotide substitutions per site (subs/site).

Supplementary Figure 4 Phylogenic tree of H5 subtype influenza viruses using HA gene sequences with available the other influenza viruses. The HA genes of H5 subtype viruses collected during 2021 in China together with the other influenza viruses were analyzed. The isolates with red color denote the H5N1, H5N6, and H5N8 viruses used in this study. The scale bar represents the number of nucleotide substitutions per site (subs/site).

Supplementary Figure 5 Phylogenic tree of H5 subtype influenza viruses using NP gene sequences with available the other influenza viruses. The NP genes of H5 subtype viruses collected during 2021 in China together with the other influenza viruses were analyzed. The isolates with red color denote the H5N1, H5N6, and H5N8 viruses used in this study. The scale bar represents the number of nucleotide substitutions per site (subs/site).

Supplementary Figure 6 Phylogenic tree of H5N1 subtype influenza viruses using NA gene sequences with available the other influenza viruses. The NA genes of H5N1 viruses collected during 2021 in China together with the other influenza viruses were analyzed. The isolates with red color denote the H5N1 viruses used in this study. The scale bar represents the number of nucleotide substitutions per site (subs/site).

Supplementary Figure 7 Phylogenic tree of H5N6 subtype influenza viruses using NA gene sequences with available the other influenza viruses. The NA genes of H5N6 viruses collected during 2021 in China together with the other influenza viruses were analyzed. The isolates with red color denote the H5N1, H5N6, and H5N8 viruses used in this study. The scale bar represents the number of nucleotide substitutions per site (subs/site).

Supplementary Figure 8 Phylogenic tree of H5N8 subtype influenza viruses using NA gene sequences with available the other influenza viruses. The NA genes of H5N8 viruses collected during 2021 in China together with the other influenza viruses were analyzed. The isolates with red color denote the H5N1, H5N6, and H5N8 viruses used in this study. The scale bar represents the number of nucleotide substitutions per site (subs/site).

Supplementary Figure 9 Phylogenic tree of H5 subtype influenza viruses using M gene sequences with available the other influenza viruses. The M genes of H5 subtype viruses collected during 2021 in China together with the other influenza viruses were analyzed. The isolates with red color denote the H5N1, H5N6, and H5N8 viruses used in this study. The scale bar represents the number of nucleotide substitutions per site (subs/site).

Supplementary Figure 10 Phylogenic tree of H5 subtype influenza viruses using NS gene sequences with available the other influenza viruses. The NS genes of H5 subtype viruses collected during 2021 in China together with the other influenza viruses were analyzed. The isolates with red color denote the H5N1, H5N6, and H5N8 viruses used in this study. The scale bar represents the number of nucleotide substitutions per site (subs/site).

Supplementary Table 1 The model of eight gene segment using IQ-TREE.

Supplementary Table 2 The accession number in GISAISD of avian influenza H5 subtype viruses used in this study.

Supplementary Table 3 Molecular features of the HA and NA protein of avian influenza A H5 subtype viruses in this study.

Supplementary Table 4 Molecular features of the PB2 genes of avian influenza A H5 subtype viruses in this study.

Supplementary Table 5 Molecular features of the PB1 protein of avian influenza A H5 subtype viruses in this study.

Supplementary Table 6 Molecular features of the PA protein of avian influenza A H5 subtype viruses in this study.

Supplementary Table 7 Molecular features of the NP protein of avian influenza A H5 subtype viruses in this study.

Supplementary Table 8 Molecular features of the M1 protein of avian influenza A H5 subtype viruses in this study.

Supplementary Table 9 Molecular features of the NS1 protein of avian influenza A H5 subtype viruses in this study.
