# Supplementary material for: Divergent Reassortment and Transmission Dynamics of Highly Pathogenic Avian Influenza A(H5N8) Virus in Birds of China During 2021
Source: Front Microbiol. 2022 Jun 30;13:913551. doi: 10.3389/fmicb.2022.913551 (PMC9279683; doi:10.3389/fmicb.2022.913551)

Supplementary Table 1. The model of eight gene segment using IQ-TREE.

**Gene segment**	**Model selection**
PB2	GTR+F+G4
PB1	GTR+F+G4
PA	K3Pu+F+I+G4
HA	GTR+F+G4
NP	GTR+F+G4
N1	K3Pu+F+G4
N6	TPM2u+F+G4
N8	K3Pu+F+G4
M	SYM+I+G44
NS	TVM+F+G4

Supplementary Table 2. The accession number in GISAISD of avian influenza H5 subtype viruses used in this study.

Isolation name	Accession Number	PB2	PB1	PA	HA	NP	NA	M	NS
A/Duck/Sichuan/21022-1/2021(H5N8)	EPI_ISL_7349517	EPI1941510	EPI1941511	EPI1941515	EPI1941509	EPI1941521	EPI1941524	EPI1941525	EPI1941526
A/Goose/Henan/21028/2021(H5N8)	EPI_ISL_7379463	EPI1941543	EPI1941544	EPI1941545	EPI1941542	EPI1941546	EPI1941547	EPI1941548	EPI1941549
A/Duck/Sichuan/21044-2/2021(H5N8)	EPI_ISL_7379464	EPI1941551	EPI1941552	EPI1941553	EPI1941550	EPI1941555	EPI1941556	EPI1941557	EPI1941558
A/Goose/Henan/21056-2/2021(H5N8)	EPI_ISL_7379465	EPI1941560	EPI1941561	EPI1941562	EPI1941559	EPI1941563	EPI1941564	EPI1941565	EPI1941566
A/Duck/Guangdong/21057/2021(H5N8)	EPI_ISL_7379509	EPI1941568	EPI1941569	EPI1941570	EPI1941567	EPI1941571	EPI1941572	EPI1941573	EPI1941574
A/Goose/Jiangsu/21153-2/2021(H5N8)	EPI_ISL_7380253	EPI1941576	EPI1941577	EPI1941578	EPI1941579	EPI1941580	EPI1941581	EPI1941582	EPI1941575
A/Goose/Shandong/21153-3/2021(H5N8)	EPI_ISL_7380500	EPI1941583	EPI1941584	EPI1941585	EPI1941586	EPI1941587	EPI1941588	EPI1941589	EPI1941590

A/Duck/Shandong/21155-1/2021(H5N8)	EPI_ISL_7380501	EPI1941591	EPI1941592	EPI1941593	EPI1941594	EPI1941595	EPI1941596	EPI1941597	EPI1941598
A/Chicken/Jiangsu/21187/2021(H5N8)	EPI_ISL_7380537	EPI1941599	EPI1941600	EPI1941601	EPI1941602	EPI1941603	EPI1941604	EPI1941605	EPI1941606
A/Chicken/Guangdong/21199-2/2021(H5N8)	EPI_ISL_7380538	EPI1941607	EPI1941608	EPI1941609	EPI1941610	EPI1941611	EPI1941612	EPI1941613	EPI1941614
A/Duck/Shandong/21232-5/2021(H5N8)	EPI_ISL_7380623	EPI1941616	EPI1941617	EPI1941618	EPI1941615	EPI1941619	EPI1941620	EPI1941621	EPI1941622
A/Duck/Guangdong/21316/2021(H5N8)	EPI_ISL_7380813	EPI1941624	EPI1941625	EPI1941626	EPI1941623	EPI1941627	EPI1941628	EPI1941629	EPI1941630
A/Chicken/Liaoning/21346-2/2021(H5N8)	EPI_ISL_7381026	EPI1941632	EPI1941633	EPI1941634	EPI1941631	EPI1941635	EPI1941636	EPI1941637	EPI1941638
A/Goose/Shandong/21369-4/2021(H5N8)	EPI_ISL_7381065	EPI1941640	EPI1941641	EPI1941642	EPI1941639	EPI1941643	EPI1941644	EPI1941645	EPI1941646
A/Goose/Shandong/21369-5/2021(H5N8)	EPI_ISL_7381103	EPI1941648	EPI1941649	EPI1941650	EPI1941647	EPI1941651	EPI1941652	EPI1941653	EPI1941654
A/Duck/Shandong/21376-1/2021(H5N8)	EPI_ISL_7381104	EPI1941656	EPI1941657	EPI1941658	EPI1941655	EPI1941659	EPI1941660	EPI1941661	EPI1941662

A/Goose/Liaoning/21640/2021 (H5N8)	EPI_ISL_738 1115	EPI1941 664	EPI1941 665	EPI1941 666	EPI1941 663	EPI1941 667	EPI1941 668	EPI1941 669	EPI1941 687
A/Duck/Shandong/21644-4/2021(H5N8)	EPI_ISL_738 1183	EPI1941 672	EPI1941 673	EPI1941 674	EPI1941 671	EPI1941 675	EPI1941 679	EPI1941 681	EPI1941 684
A/Duck/Shandong/21644-7/2021(H5N8)	EPI_ISL_738 1397	EPI1941 676	EPI1941 677	EPI1941 678	EPI1941 680	EPI1941 682	EPI1941 683	EPI1941 685	EPI1941 686
A/Goose/Liaoning/21723/2021 (H5N8)	EPI_ISL_738 1413	EPI1941 688	EPI1941 689	EPI1941 690	EPI1941 691	EPI1941 692	EPI1941 693	EPI1941 694	EPI1941 695
A/Duck/Shandong/21931-8/2021(H5N8)	EPI_ISL_738 1423	EPI1941 696	EPI1941 697	EPI1941 698	EPI1941 699	EPI1941 700	EPI1941 701	EPI1941 702	EPI1941 703
A/Duck/Guangdong/21964/2021(H5N8)	EPI_ISL_738 1438	EPI1941 704	EPI1941 705	EPI1941 706	EPI1941 707	EPI1941 708	EPI1941 709	EPI1941 710	EPI1941 711
A/Chicken/Guangxi/21989-3/2021(H5N8)	EPI_ISL_738 1448	EPI1941 712	EPI1941 713	EPI1941 714	EPI1941 715	EPI1941 716	EPI1941 717	EPI1941 718	EPI1941 719
A/Goose/Guangdong/211030-1/2021(H5N8)	EPI_ISL_738 1695	EPI1941 720	EPI1941 721	EPI1941 722	EPI1941 723	EPI1941 724	EPI1941 725	EPI1941 726	EPI1941 727
A/Duck/Shandong/21232-2/2021(H5N1)	EPI_ISL_740 4975	EPI1942 112	EPI1942 113	EPI1942 114	EPI1942 274	EPI1942 115	EPI1942 116	EPI1942 117	EPI1942 118

A/Duck/Shandong/21376-4/2021(H5N1)	EPI_ISL_7405093	EPI1942119	EPI1942120	EPI1942121	EPI1942122	EPI1942123	EPI1942124	EPI1942125	EPI1942126
A/Duck/Shandong/21931-6/2021(H5N1)	EPI_ISL_7405231	EPI1942127	EPI1942128	EPI1942129	EPI1942130	EPI1942131	EPI1942132	EPI1942133	EPI1942134
A/Chicken/Guangdong/211106-1/2021(H5N6)	EPI_ISL_7457547	EPI1942194	EPI1942195	EPI1942196	EPI1942197	EPI1942198	EPI1942199	EPI1942200	EPI1942201
A/Chicken/Guangdong/211106-3/2021(H5N6)	EPI_ISL_7457654	EPI1942202	EPI1942203	EPI1942204	EPI1942205	EPI1942206	EPI1942207	EPI1942208	EPI1942209
A/Duck/Shandong/21376-5/2021(H5N8)	EPI_ISL_7457708	EPI1942210	EPI1942379	EPI1942211	EPI1942212	EPI1942213	EPI1942214	EPI1942215	EPI1942216
A/Duck/Shandong/21644-1/2021(H5N8)	EPI_ISL_7458702	EPI1942231	EPI1942232	EPI1942233	EPI1942230	EPI1942234	EPI1942235	EPI1942236	EPI1942370
A/Goose/Guangdong/21858/2021(H5N8)	EPI_ISL_7459886	EPI1942245	EPI1942380	EPI1942246	EPI1942244	EPI1942247	EPI1942248	EPI1942249	EPI1942250
A/Duck/Anhui/21931-1/2021(H5N8)	EPI_ISL_7459891	EPI1942252	EPI1942381	EPI1942253	EPI1942251	EPI1942254	EPI1942255	EPI1942256	EPI1942257
A/Duck/Shandong/21931-3/2021(H5N8)	EPI_ISL_7459900	EPI1942259	EPI1942384	EPI1942260	EPI1942258	EPI1942261	EPI1942262	EPI1942263	EPI1942264

A/Duck/Sichuan/21022-4/2021(H5N8)	EPI_ISL_7192735	EPI1940243	EPI1940244	EPI1940245	EPI1940246	EPI1942275	EPI1940247	EPI1940248	EPI1940249

Supplementary Table 3. Molecular features of the HA and NA protein of avian influenza A H5 subtype viruses in this study.

Isolates	HA											
	**D94N**	**S121N**	**S133A**	**A134V**	**G139R**	**S154N**	**S155N**	**T156A**	**N182K/D**	**D183G**	**E186G**	**T188I**
21022-1	S	S	A	A	G	N	D	A	N	N	E	I
21028	S	S	A	A	G	N	D	A	N	N	E	I
21044-2	S	S	A	A	G	N	D	A	N	N	E	I
21056-2	S	S	A	A	G	N	D	A	N	N	E	I
21057	S	S	A	A	G	N	D	A	N	N	E	I
21153-2	S	S	A	A	G	N	D	A	N	N	E	I
21153-3	S	S	A	A	G	N	D	A	N	N	E	I
21155-1	S	S	A	A	G	N	D	A	N	N	E	I
21187	S	S	A	A	G	N	D	A	N	N	E	I
21199-2	S	S	A	A	G	N	D	A	N	N	E	I
21232-5	S	S	A	A	G	N	D	A	N	N	E	I
21316	S	S	A	A	G	N	D	A	N	N	E	I
21346-2	S	S	A	A	G	N	D	A	N	N	E	I
21369-4	S	S	A	A	G	N	D	A	N	N	E	I
21369-5	S	S	A	A	G	N	D	A	N	N	E	I

21376-1	S	S	A	A	G	N	D	A	N	N	E	I
21640	S	S	A	A	G	N	D	A	N	N	E	I
21644-4	S	S	A	A	G	N	D	A	N	N	E	I
21644-7	S	S	A	A	G	N	D	A	N	N	E	I
21723	S	S	A	A	G	N	D	A	N	N	E	I
21931-8	S	S	A	A	G	N	D	A	N	N	E	I
21964	S	S	A	A	G	N	N	A	N	N	E	M
21989-3	S	S	A	A	G	N	N	A	N	N	E	I
211030-1	N	S	A	A	G	N	D	A	N	N	E	I
21232-2	S	S	A	A	G	N	D	A	N	N	E	I
21376-4	S	S	A	A	G	S	D	A	N	N	E	I
21931-6	S	S	A	A	G	N	D	A	N	N	E	I
211106-1	S	S	A	A	G	N	D	A	N	N	E	I
211106-3	S	S	A	A	G	N	D	A	N	N	E	I
21376-5	S	S	A	A	G	N	D	A	N	N	E	T
21644-1	S	S	A	A	G	N	D	A	N	N	E	I
21858	S	S	A	A	G	N	D	A	N	N	E	I
21931-1	S	S	A	A	G	N	D	A	N	N	E	I
21931-3	S	S	A	A	G	N	D	A	N	N	E	I

21022-4	S	S	A	A	G	N	D	A	N	N	E	I

**Isolates**	**HA**											**NA**
	**K189R/T**	**Q192R/H**	**N193K**	**V210I**	**K218Q**	**G221D**	**Q222L**	**S223N**	**G224S**	**P235S**	**E251K**	**N294S**
21022-1	N	K	N	V	Q	G	Q	R	G	P	E	N
21028	N	K	N	V	Q	G	Q	R	G	P	E	N
21044-2	N	K	N	V	Q	G	Q	R	G	P	E	N
21056-2	N	K	N	V	Q	G	Q	R	G	P	E	N
21057	N	K	N	V	Q	G	Q	R	G	P	E	N
21153-2	N	K	N	V	Q	G	Q	R	G	P	E	N
21153-3	N	K	N	V	Q	G	Q	R	G	P	E	N
21155-1	N	K	N	V	Q	G	Q	R	G	P	E	N
21187	N	K	N	V	Q	G	Q	R	G	P	E	N
21199-2	N	K	N	V	Q	G	Q	R	G	P	E	N
21232-5	N	K	N	V	Q	G	Q	R	G	P	E	N
21316	N	K	N	V	Q	G	Q	R	G	P	E	N
21346-2	N	K	N	V	Q	G	Q	R	G	P	E	N
21369-4	N	K	N	V	Q	G	Q	R	G	P	E	N
21369-5	N	K	N	V	Q	G	Q	R	G	P	E	N

21376-1	N	K	N	V	Q	G	Q	R	G	P	E	N
21640	N	K	N	V	Q	G	Q	R	G	P	E	N
21644-4	N	K	N	V	Q	G	Q	R	G	P	E	N
21644-7	N	K	N	V	Q	G	Q	R	G	P	E	N
21723	N	K	N	V	Q	G	Q	R	G	P	E	N
21931-8	N	K	N	V	Q	G	Q	R	G	P	E	N
21964	N	K	N	V	Q	G	Q	R	G	P	E	N
21989-3	N	K	N	V	Q	G	Q	R	G	P	E	N
211030-1	N	K	N	V	Q	G	Q	R	G	P	E	N
21232-2	N	K	N	V	Q	G	Q	R	G	P	E	N
21376-4	N	K	N	V	Q	G	Q	R	G	P	E	N
21931-6	N	K	N	V	Q	G	Q	R	G	P	E	N
211106-1	N	K	N	V	Q	G	Q	R	G	P	E	N
211106-3	N	K	N	V	Q	G	Q	R	G	P	E	N
21376-5	N	K	N	V	Q	G	Q	R	G	P	E	N
21644-1	N	K	N	V	Q	G	Q	R	G	P	E	N
21858	N	K	N	V	Q	G	Q	R	G	P	E	N
21931-1	N	K	N	V	Q	G	Q	R	G	P	E	N
21931-3	N	K	N	V	Q	G	Q	R	G	P	E	N

21022-4	N	K	N	V	Q	G	Q	R	G	P	E	N

A/Duck/Sichuan/21022-1/2021(H5N8), 21022-1; A/Goose/Henan/21028/2021(H5N8), 21028; A/Duck/Sichuan/21044-2/2021(H5N8), 21044-2; A/Goose/Henan/21056-2/2021(H5N8), 21056-2; A/Duck/Guangdong/21057/2021(H5N8), 21057; A/Goose/Jiangsu/21153-2/2021(H5N8), 21153-2; A/Goose/Shandong/21153-3/2021(H5N8), 21153-3; A/Duck/Shandong/21155-1/2021(H5N8), 21155-1; A/Chicken/Jiangsu/21187/2021(H5N8), 21187; A/Chicken/Guangdong/21199-2/2021(H5N8), 21199-2; A/Duck/Shandong/21232-5/2021(H5N8), 21232-5; A/Duck/Guangdong/21316/2021(H5N8), 21316; A/Chicken/Liaoning/21346-2/2021(H5N8), 21346-2; A/Goose/Shandong/21369-4/2021(H5N8), 21369-4; A/Goose/Shandong/21369-5/2021(H5N8), 21369-5; A/Duck/Shandong/21376-1/2021(H5N8), 21376-1; A/Goose/Liaoning/21640/2021(H5N8), 21640; A/Duck/Shandong/21644-4/2021(H5N8), 21644-4; A/Duck/Shandong/21644-7/2021(H5N8), 21644-7; A/Goose/Liaoning/21723/2021(H5N8), 21723; A/Duck/Shandong/21931-8/2021(H5N8), 21931-8; A/Duck/Guangdong/21964/2021(H5N8), 21964; A/Chicken/Guangxi/21989-3/2021(H5N8), 21989-3; A/Goose/Guangdong/211030-1/2021(H5N8), 211030-1; A/Duck/Shandong/21232-2/2021(H5N1), 21232-2; A/Duck/Shandong/21376-4/2021(H5N1), 21376-4; A/Duck/Shandong/21931-6/2021(H5N1), 21931-6; A/Chicken/Guangdong/211106-1/2021(H5N6), 211106-1; A/Chicken/Guangdong/211106-3/2021(H5N6), 211106-3; A/Duck/Shandong/21376-5/2021(H5N8), 21376-5; A/Duck/Shandong/21644-1/2021(H5N8), 21644-1;

A/Goose/Guangdong/21858/2021(H5N8), 21858; A/Duck/Anhui/21931-1/2021(H5N8), 21931-1; A/Duck/Shandong/21931-3/2021(H5N8), 21931-3; A/Duck/Sichuan/21022-4/2021(H5N8), 21022-4. HA numbering relative to A/Vietnam/1203/2004(H5N1).

Supplementary Table 4. Molecular features of the PB2 genes of avian influenza A H5 subtype viruses in this study.

Viruses	D9V	V25A	E158G	E192K	A199S	D256G	K526R	Q591K	A588V	E627K	D701N
21022-1	D	V	E	E	A	D	K	Q	A	E	D
21028	D	V	E	E	A	D	K	Q	A	E	D
21044-2	D	V	E	E	A	D	K	Q	A	E	D
21056-2	D	V	E	E	A	D	K	Q	A	E	D
21057	D	V	E	E	A	D	K	Q	A	E	D
21153-2	D	V	E	E	A	D	K	Q	A	E	D
21153-3	D	V	E	E	A	D	K	Q	A	E	D
21155-1	D	V	E	E	A	D	K	Q	A	E	D
21187	D	V	E	E	A	D	K	Q	A	E	D
21199-2	D	V	E	E	A	D	K	Q	A	E	D
21232-5	D	V	E	E	A	D	K	Q	A	E	D
21316	D	V	E	E	A	D	K	Q	A	E	D
21346-2	D	V	E	E	A	D	K	Q	A	E	D
21369-4	D	V	E	E	A	D	K	Q	A	E	D
21369-5	D	V	E	E	A	D	K	Q	A	E	D
21376-1	D	V	E	E	A	D	K	Q	A	E	D

21640	D	V	E	E	A	D	K	Q	A	E	D
21644-4	D	V	E	E	A	D	K	Q	A	E	D
21644-7	D	V	E	E	A	D	K	Q	A	E	D
21723	D	V	E	E	A	D	K	Q	A	E	D
21931-8	D	V	K	E	A	D	K	Q	A	E	D
21964	D	V	K	E	A	D	K	Q	A	E	D
21989-3	D	V	K	E	A	D	K	Q	A	E	D
211030-1	D	V	K	E	A	D	K	Q	A	E	D
21232-2	D	V	E	E	A	D	K	Q	A	E	D
21376-4	D	V	E	E	A	D	K	Q	A	E	D
21931-6	D	V	K	E	A	D	K	Q	A	E	D
211106-1	D	V	E	E	A	D	K	Q	A	E	D
211106-3	D	V	E	E	A	D	K	Q	A	E	D
21376-5	D	V	E	E	A	D	K	Q	A	E	D
21644-1	D	V	E	E	A	D	K	Q	A	E	D
21858	D	V	E	E	A	D	K	Q	A	E	D
21931-1	D	V	K	E	A	D	K	Q	A	E	D
21931-3	D	V	K	E	A	D	K	Q	A	E	D
21022-4	D	V	E	E	A	D	K	Q	A	E	D

A/Duck/Sichuan/21022-1/2021(H5N8), 21022-1; A/Goose/Henan/21028/2021(H5N8), 21028; A/Duck/Sichuan/21044-2/2021(H5N8), 21044-2; A/Goose/Henan/21056-2/2021(H5N8), 21056-2; A/Duck/Guangdong/21057/2021(H5N8), 21057; A/Goose/Jiangsu/21153-2/2021(H5N8), 21153-2; A/Goose/Shandong/21153-3/2021(H5N8), 21153-3; A/Duck/Shandong/21155-1/2021(H5N8), 21155-1; A/Chicken/Jiangsu/21187/2021(H5N8), 21187; A/Chicken/Guangdong/21199-2/2021(H5N8), 21199-2; A/Duck/Shandong/21232-5/2021(H5N8), 21232-5; A/Duck/Guangdong/21316/2021(H5N8), 21316; A/Chicken/Liaoning/21346-2/2021(H5N8), 21346-2; A/Goose/Shandong/21369-4/2021(H5N8), 21369-4; A/Goose/Shandong/21369-5/2021(H5N8), 21369-5; A/Duck/Shandong/21376-1/2021(H5N8), 21376-1; A/Goose/Liaoning/21640/2021(H5N8), 21640; A/Duck/Shandong/21644-4/2021(H5N8), 21644-4; A/Duck/Shandong/21644-7/2021(H5N8), 21644-7; A/Goose/Liaoning/21723/2021(H5N8), 21723; A/Duck/Shandong/21931-8/2021(H5N8), 21931-8; A/Duck/Guangdong/21964/2021(H5N8), 21964; A/Chicken/Guangxi/21989-3/2021(H5N8), 21989-3; A/Goose/Guangdong/211030-1/2021(H5N8), 211030-1; A/Duck/Shandong/21232-2/2021(H5N1), 21232-2; A/Duck/Shandong/21376-4/2021(H5N1), 21376-4; A/Duck/Shandong/21931-6/2021(H5N1), 21931-6; A/Chicken/Guangdong/211106-1/2021(H5N6), 211106-1; A/Chicken/Guangdong/211106-3/2021(H5N6), 211106-3; A/Duck/Shandong/21376-5/2021(H5N8), 21376-5; A/Duck/Shandong/21644-1/2021(H5N8), 21644-1;

A/Goose/Guangdong/21858/2021(H5N8), 21858; A/Duck/Anhui/21931-1/2021(H5N8), 21931-1; A/Duck/Shandong/21931-3/2021(H5N8), 21931-3; A/Duck/Sichuan/21022-4/2021(H5N8), 21022-4.

Supplementary Table 5. Molecular features of the PB1 protein of avian influenza A H5 subtype viruses in this study.

Isolates	D3V	H99Y	N105S	D622G	T677M
21022-1	V	H	N	G	T
21028	V	H	I	G	T
21044-2	V	H	N	G	T
21056-2	V	H	N	G	T
21057	V	H	I	G	T
21153-2	V	H	N	G	T
21153-3	V	H	N	G	T
21155-1	V	H	N	G	T
21187	V	H	N	G	T
21199-2	V	H	N	G	T
21232-5	V	H	N	G	T
21316	V	H	N	G	T
21346-2	V	H	N	G	T
21369-4	V	H	N	G	T
21369-5	V	H	N	G	T
21376-1	V	H	N	G	T

21640	V	H	N	G	T
21644-4	V	H	N	G	T
21644-7	V	H	N	G	T
21723	V	H	N	G	T
21931-8	V	H	N	G	T
21964	V	H	N	G	T
21989-3	V	H	N	G	T
211030-1	V	H	N	G	T
21232-2	V	H	N	G	T
21376-4	V	H	N	G	T
21931-6	V	H	N	G	T
211106-1	C	H	N	G	T
211106-3	C	H	N	G	T
21376-5	V	H	N	G	T
21644-1	V	H	N	G	T
21858	V	H	N	G	T
21931-1	V	H	N	G	T
21931-3	V	H	N	G	T
21022-4	V	H	N	G	T

A/Duck/Sichuan/21022-1/2021(H5N8), 21022-1; A/Goose/Henan/21028/2021(H5N8), 21028; A/Duck/Sichuan/21044-2/2021(H5N8), 21044-2; A/Goose/Henan/21056-2/2021(H5N8), 21056-2; A/Duck/Guangdong/21057/2021(H5N8), 21057; A/Goose/Jiangsu/21153-2/2021(H5N8), 21153-2; A/Goose/Shandong/21153-3/2021(H5N8), 21153-3; A/Duck/Shandong/21155-1/2021(H5N8), 21155-1; A/Chicken/Jiangsu/21187/2021(H5N8), 21187; A/Chicken/Guangdong/21199-2/2021(H5N8), 21199-2; A/Duck/Shandong/21232-5/2021(H5N8), 21232-5; A/Duck/Guangdong/21316/2021(H5N8), 21316; A/Chicken/Liaoning/21346-2/2021(H5N8), 21346-2; A/Goose/Shandong/21369-4/2021(H5N8), 21369-4; A/Goose/Shandong/21369-5/2021(H5N8), 21369-5; A/Duck/Shandong/21376-1/2021(H5N8), 21376-1; A/Goose/Liaoning/21640/2021(H5N8), 21640; A/Duck/Shandong/21644-4/2021(H5N8), 21644-4; A/Duck/Shandong/21644-7/2021(H5N8), 21644-7; A/Goose/Liaoning/21723/2021(H5N8), 21723; A/Duck/Shandong/21931-8/2021(H5N8), 21931-8; A/Duck/Guangdong/21964/2021(H5N8), 21964; A/Chicken/Guangxi/21989-3/2021(H5N8), 21989-3; A/Goose/Guangdong/211030-1/2021(H5N8), 211030-1; A/Duck/Shandong/21232-2/2021(H5N1), 21232-2; A/Duck/Shandong/21376-4/2021(H5N1), 21376-4; A/Duck/Shandong/21931-6/2021(H5N1), 21931-6; A/Chicken/Guangdong/211106-1/2021(H5N6), 211106-1; A/Chicken/Guangdong/211106-3/2021(H5N6), 211106-3; A/Duck/Shandong/21376-5/2021(H5N8), 21376-5; A/Duck/Shandong/21644-1/2021(H5N8), 21644-1;

A/Goose/Guangdong/21858/2021(H5N8), 21858; A/Duck/Anhui/21931-1/2021(H5N8), 21931-1; A/Duck/Shandong/21931-3/2021(H5N8), 21931-3; A/Duck/Sichuan/21022-4/2021(H5N8), 21022-4. HA numbering relative to A/Vietnam/1203/2004(H5N1).

Supplementary Table 6. Molecular features of the PA protein of avian influenza A H5 subtype viruses in this study.

Isolates	T97I	K142N	K159R	N383D	S421I	R443K
21022-1	T	R	A	D	S	R
21028	T	K	A	D	S	R
21044-2	T	R	A	D	S	R
21056-2	T	K	A	D	S	R
21057	T	K	A	D	S	R
21153-2	T	K	A	D	S	R
21153-3	T	K	A	D	S	R
21155-1	T	K	A	D	S	R
21187	T	K	A	D	S	R
21199-2	T	K	A	D	S	R
21232-5	T	K	A	D	S	R
21316	T	K	A	D	S	R
21346-2	T	K	A	D	S	R
21369-4	T	K	A	D	S	R
21369-5	T	K	A	D	S	R
21376-1	T	K	A	D	S	R

21640	T	K	A	D	S	R
21644-4	T	K	A	D	S	R
21644-7	T	K	A	D	G	R
21723	T	K	A	D	S	R
21931-8	T	K	A	D	S	R
21964	T	K	A	D	S	R
21989-3	T	K	A	D	S	R
211030-1	T	K	A	D	S	R
21232-2	T	K	A	D	S	R
21376-4	T	K	A	D	S	R
21931-6	T	K	A	D	S	R
211106-1	T	K	A	D	S	R
211106-3	T	K	A	D	S	R
21376-5	T	K	A	D	S	R
21644-1	T	K	A	D	S	R
21858	T	K	A	D	S	R
21931-1	T	K	A	D	S	R
21931-3	T	K	A	D	S	R
21022-4	T	R	A	D	S	R

A/Duck/Sichuan/21022-1/2021(H5N8), 21022-1; A/Goose/Henan/21028/2021(H5N8), 21028; A/Duck/Sichuan/21044-2/2021(H5N8), 21044-2; A/Goose/Henan/21056-2/2021(H5N8), 21056-2; A/Duck/Guangdong/21057/2021(H5N8), 21057; A/Goose/Jiangsu/21153-2/2021(H5N8), 21153-2; A/Goose/Shandong/21153-3/2021(H5N8), 21153-3; A/Duck/Shandong/21155-1/2021(H5N8), 21155-1; A/Chicken/Jiangsu/21187/2021(H5N8), 21187; A/Chicken/Guangdong/21199-2/2021(H5N8), 21199-2; A/Duck/Shandong/21232-5/2021(H5N8), 21232-5; A/Duck/Guangdong/21316/2021(H5N8), 21316; A/Chicken/Liaoning/21346-2/2021(H5N8), 21346-2; A/Goose/Shandong/21369-4/2021(H5N8), 21369-4; A/Goose/Shandong/21369-5/2021(H5N8), 21369-5; A/Duck/Shandong/21376-1/2021(H5N8), 21376-1; A/Goose/Liaoning/21640/2021(H5N8), 21640; A/Duck/Shandong/21644-4/2021(H5N8), 21644-4; A/Duck/Shandong/21644-7/2021(H5N8), 21644-7; A/Goose/Liaoning/21723/2021(H5N8), 21723; A/Duck/Shandong/21931-8/2021(H5N8), 21931-8; A/Duck/Guangdong/21964/2021(H5N8), 21964; A/Chicken/Guangxi/21989-3/2021(H5N8), 21989-3; A/Goose/Guangdong/211030-1/2021(H5N8), 211030-1; A/Duck/Shandong/21232-2/2021(H5N1), 21232-2; A/Duck/Shandong/21376-4/2021(H5N1), 21376-4; A/Duck/Shandong/21931-6/2021(H5N1), 21931-6; A/Chicken/Guangdong/211106-1/2021(H5N6), 211106-1; A/Chicken/Guangdong/211106-3/2021(H5N6), 211106-3; A/Duck/Shandong/21376-5/2021(H5N8), 21376-5; A/Duck/Shandong/21644-1/2021(H5N8), 21644-1;

A/Goose/Guangdong/21858/2021(H5N8), 21858; A/Duck/Anhui/21931-1/2021(H5N8), 21931-1; A/Duck/Shandong/21931-3/2021(H5N8), 21931-3; A/Duck/Sichuan/21022-4/2021(H5N8), 21022-4.

Supplementary Table 7. Molecular features of the NP protein of avian influenza A H5 subtype viruses in this study.

Isolates	M105V	I109T	A184K	K227R	K229R	N319K	K470R
21022-1	V	I	K	K	K	N	K
21028	V	I	K	K	K	N	K
21044-2	V	I	K	K	K	N	K
21056-2	V	I	K	K	K	N	K
21057	V	I	K	K	K	N	K
21153-2	V	I	K	K	K	N	K
21153-3	V	I	K	K	K	N	K
21155-1	V	I	K	K	K	N	K
21187	V	I	K	K	K	N	K
21199-2	V	I	K	K	K	N	K
21232-5	V	I	K	K	K	N	K
21316	V	I	K	K	K	N	K
21346-2	V	I	K	K	K	N	K
21369-4	V	I	K	K	K	N	K
21369-5	V	I	K	K	K	N	K
21376-1	V	I	K	K	K	N	K

21640	V	I	K	K	K	N	K
21644-4	V	I	K	K	K	N	K
21644-7	V	I	K	K	K	N	K
21723	V	I	K	K	K	N	K
21931-8	V	I	K	K	K	N	K
21964	V	I	K	K	K	N	K
21989-3	V	I	K	K	K	N	K
211030-1	M	I	K	K	K	N	K
21232-2	M	I	K	K	K	N	K
21376-4	M	I	K	K	K	N	K
21931-6	M	I	K	K	K	N	K
211106-1	V	I	K	K	K	N	K
211106-3	V	I	K	K	K	N	K
21376-5	V	I	K	K	K	N	K
21644-1	V	I	K	K	K	N	K
21858	V	I	K	K	K	N	K
21931-1	V	I	K	K	K	N	K
21931-3	V	I	K	K	K	N	K
21022-4	V	I	K	K	K	N	K

A/Duck/Sichuan/21022-1/2021(H5N8), 21022-1; A/Goose/Henan/21028/2021(H5N8), 21028; A/Duck/Sichuan/21044-2/2021(H5N8), 21044-2; A/Goose/Henan/21056-2/2021(H5N8), 21056-2; A/Duck/Guangdong/21057/2021(H5N8), 21057; A/Goose/Jiangsu/21153-2/2021(H5N8), 21153-2; A/Goose/Shandong/21153-3/2021(H5N8), 21153-3; A/Duck/Shandong/21155-1/2021(H5N8), 21155-1; A/Chicken/Jiangsu/21187/2021(H5N8), 21187; A/Chicken/Guangdong/21199-2/2021(H5N8), 21199-2; A/Duck/Shandong/21232-5/2021(H5N8), 21232-5; A/Duck/Guangdong/21316/2021(H5N8), 21316; A/Chicken/Liaoning/21346-2/2021(H5N8), 21346-2; A/Goose/Shandong/21369-4/2021(H5N8), 21369-4; A/Goose/Shandong/21369-5/2021(H5N8), 21369-5; A/Duck/Shandong/21376-1/2021(H5N8), 21376-1; A/Goose/Liaoning/21640/2021(H5N8), 21640; A/Duck/Shandong/21644-4/2021(H5N8), 21644-4; A/Duck/Shandong/21644-7/2021(H5N8), 21644-7; A/Goose/Liaoning/21723/2021(H5N8), 21723; A/Duck/Shandong/21931-8/2021(H5N8), 21931-8; A/Duck/Guangdong/21964/2021(H5N8), 21964; A/Chicken/Guangxi/21989-3/2021(H5N8), 21989-3; A/Goose/Guangdong/211030-1/2021(H5N8), 211030-1; A/Duck/Shandong/21232-2/2021(H5N1), 21232-2; A/Duck/Shandong/21376-4/2021(H5N1), 21376-4; A/Duck/Shandong/21931-6/2021(H5N1), 21931-6; A/Chicken/Guangdong/211106-1/2021(H5N6), 211106-1; A/Chicken/Guangdong/211106-3/2021(H5N6), 211106-3; A/Duck/Shandong/21376-5/2021(H5N8), 21376-5; A/Duck/Shandong/21644-1/2021(H5N8), 21644-1;

A/Goose/Guangdong/21858/2021(H5N8), 21858; A/Duck/Anhui/21931-1/2021(H5N8), 21931-1; A/Duck/Shandong/21931-3/2021(H5N8), 21931-3; A/Duck/Sichuan/21022-4/2021(H5N8), 21022-4.

Supplementary Table 8. Molecular features of the M1 protein of avian influenza A H5 subtype viruses in this study.

Isolates	N30D	I43M	T139A	T215A
21022-1	D	M	T	A
21028	D	M	T	A
21044-2	D	M	T	A
21056-2	D	M	T	A
21057	D	M	T	A
21153-2	D	M	T	A
21153-3	D	M	T	A
21155-1	D	M	T	A
21187	D	M	T	A
21199-2	D	M	T	A
21232-5	D	M	T	A
21316	D	M	T	A
21346-2	D	M	T	A
21369-4	D	M	T	A
21369-5	D	M	T	A
21376-1	D	M	T	A

21640	D	M	T	A
21644-4	D	M	T	A
21644-7	D	M	T	A
21723	D	M	T	A
21931-8	D	M	T	A
21964	D	M	T	A
21989-3	D	M	T	A
211030-1	D	M	T	A
21232-2	D	M	T	A
21376-4	D	M	T	A
21931-6	D	M	T	A
211106-1	D	M	T	A
211106-3	D	M	T	A
21376-5	D	M	T	A
21644-1	D	M	T	A
21858	D	M	T	A
21931-1	D	M	T	A
21931-3	D	M	T	A
21022-4	D	M	T	A

A/Duck/Sichuan/21022-1/2021(H5N8), 21022-1; A/Goose/Henan/21028/2021(H5N8), 21028; A/Duck/Sichuan/21044-2/2021(H5N8), 21044-2; A/Goose/Henan/21056-2/2021(H5N8), 21056-2; A/Duck/Guangdong/21057/2021(H5N8), 21057; A/Goose/Jiangsu/21153-2/2021(H5N8), 21153-2; A/Goose/Shandong/21153-3/2021(H5N8), 21153-3; A/Duck/Shandong/21155-1/2021(H5N8), 21155-1; A/Chicken/Jiangsu/21187/2021(H5N8), 21187; A/Chicken/Guangdong/21199-2/2021(H5N8), 21199-2; A/Duck/Shandong/21232-5/2021(H5N8), 21232-5; A/Duck/Guangdong/21316/2021(H5N8), 21316; A/Chicken/Liaoning/21346-2/2021(H5N8), 21346-2; A/Goose/Shandong/21369-4/2021(H5N8), 21369-4; A/Goose/Shandong/21369-5/2021(H5N8), 21369-5; A/Duck/Shandong/21376-1/2021(H5N8), 21376-1; A/Goose/Liaoning/21640/2021(H5N8), 21640; A/Duck/Shandong/21644-4/2021(H5N8), 21644-4; A/Duck/Shandong/21644-7/2021(H5N8), 21644-7; A/Goose/Liaoning/21723/2021(H5N8), 21723; A/Duck/Shandong/21931-8/2021(H5N8), 21931-8; A/Duck/Guangdong/21964/2021(H5N8), 21964; A/Chicken/Guangxi/21989-3/2021(H5N8), 21989-3; A/Goose/Guangdong/211030-1/2021(H5N8), 211030-1; A/Duck/Shandong/21232-2/2021(H5N1), 21232-2; A/Duck/Shandong/21376-4/2021(H5N1), 21376-4; A/Duck/Shandong/21931-6/2021(H5N1), 21931-6; A/Chicken/Guangdong/211106-1/2021(H5N6), 211106-1; A/Chicken/Guangdong/211106-3/2021(H5N6), 211106-3; A/Duck/Shandong/21376-5/2021(H5N8), 21376-5; A/Duck/Shandong/21644-1/2021(H5N8), 21644-1;

A/Goose/Guangdong/21858/2021(H5N8), 21858; A/Duck/Anhui/21931-1/2021(H5N8), 21931-1; A/Duck/Shandong/21931-3/2021(H5N8), 21931-3; A/Duck/Sichuan/21022-4/2021(H5N8), 21022-4.

Supplementary Table 9. Molecular features of the NS1 protein of avian influenza A H5 subtype viruses in this study.

Isolates	80-84 deletion	D92E	N200S	G205R	V149A
21022-1	No	D	N	G	A
21028	No	D	N	G	A
21044-2	No	D	N	G	A
21056-2	No	D	N	G	A
21057	No	D	N	G	A
21153-2	No	D	N	G	A
21153-3	No	D	N	G	A
21155-1	No	D	N	G	A
21187	No	D	N	G	A
21199-2	No	D	N	G	A
21232-5	No	D	N	G	A
21316	No	D	N	G	A
21346-2	No	D	N	G	A
21369-4	No	D	N	G	A
21369-5	No	D	N	G	A

21376-1	No	D	N	G	A
21640	No	D	N	G	A
21644-4	No	D	N	G	A
21644-7	No	D	N	G	A
21723	No	D	N	G	A
21931-8	No	D	N	G	A
21964	No	D	N	G	A
21989-3	No	D	N	G	A
211030-1	No	D	N	G	A
21232-2	No	D	N	G	A
21376-4	No	D	N	G	A
21931-6	No	D	N	G	A
211106-1	No	D	N	G	A
211106-3	No	D	N	G	A
21376-5	No	D	N	G	A
21644-1	No	D	N	G	A
21858	No	D	N	G	A
21931-1	No	D	N	G	A
21931-3	No	D	N	G	A

21022-4	No	D	N	G	A

A/Duck/Sichuan/21022-1/2021(H5N8), 21022-1; A/Goose/Henan/21028/2021(H5N8), 21028; A/Duck/Sichuan/21044-2/2021(H5N8), 21044-2; A/Goose/Henan/21056-2/2021(H5N8), 21056-2; A/Duck/Guangdong/21057/2021(H5N8), 21057; A/Goose/Jiangsu/21153-2/2021(H5N8), 21153-2; A/Goose/Shandong/21153-3/2021(H5N8), 21153-3; A/Duck/Shandong/21155-1/2021(H5N8), 21155-1; A/Chicken/Jiangsu/21187/2021(H5N8), 21187; A/Chicken/Guangdong/21199-2/2021(H5N8), 21199-2; A/Duck/Shandong/21232-5/2021(H5N8), 21232-5; A/Duck/Guangdong/21316/2021(H5N8), 21316; A/Chicken/Liaoning/21346-2/2021(H5N8), 21346-2; A/Goose/Shandong/21369-4/2021(H5N8), 21369-4; A/Goose/Shandong/21369-5/2021(H5N8), 21369-5; A/Duck/Shandong/21376-1/2021(H5N8), 21376-1; A/Goose/Liaoning/21640/2021(H5N8), 21640; A/Duck/Shandong/21644-4/2021(H5N8), 21644-4; A/Duck/Shandong/21644-7/2021(H5N8), 21644-7; A/Goose/Liaoning/21723/2021(H5N8), 21723; A/Duck/Shandong/21931-8/2021(H5N8), 21931-8; A/Duck/Guangdong/21964/2021(H5N8), 21964; A/Chicken/Guangxi/21989-3/2021(H5N8), 21989-3; A/Goose/Guangdong/211030-1/2021(H5N8), 211030-1; A/Duck/Shandong/21232-2/2021(H5N1), 21232-2; A/Duck/Shandong/21376-4/2021(H5N1), 21376-4; A/Duck/Shandong/21931-6/2021(H5N1), 21931-6; A/Chicken/Guangdong/211106-1/2021(H5N6), 211106-1; A/Chicken/Guangdong/211106-

3/2021(H5N6), 211106-3; A/Duck/Shandong/21376-5/2021(H5N8), 21376-5; A/Duck/Shandong/21644-1/2021(H5N8), 21644-1; A/Goose/Guangdong/21858/2021(H5N8), 21858; A/Duck/Anhui/21931-1/2021(H5N8), 21931-1; A/Duck/Shandong/21931-3/2021(H5N8), 21931-3; A/Duck/Sichuan/21022-4/2021(H5N8), 21022-4.

Supplementary Figure 1

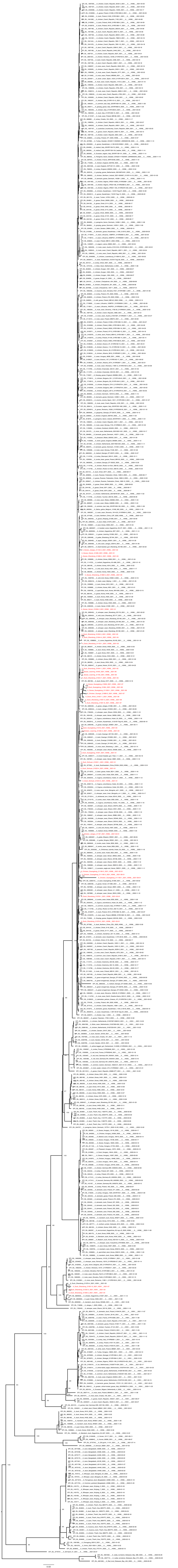

Supplementary Figure 2

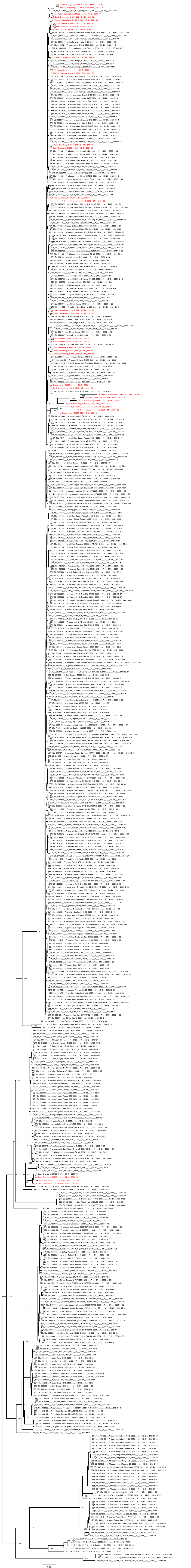

Supplementary Figure 3

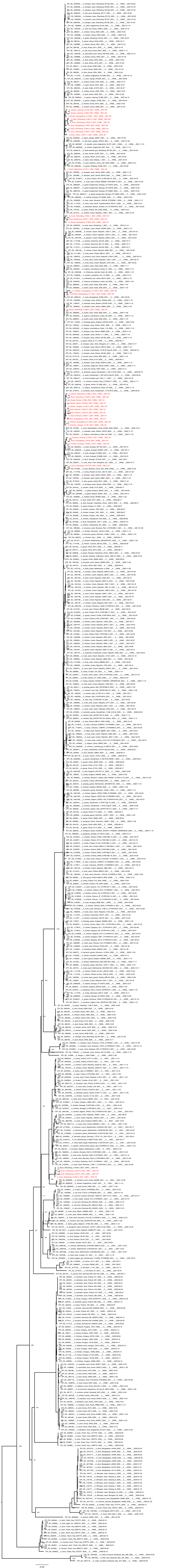

Supplementary Figure 4

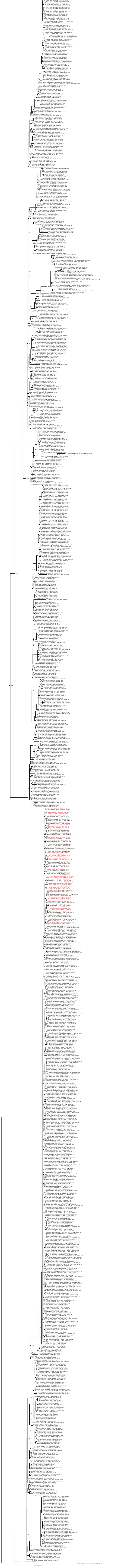

Supplementary Figure 5

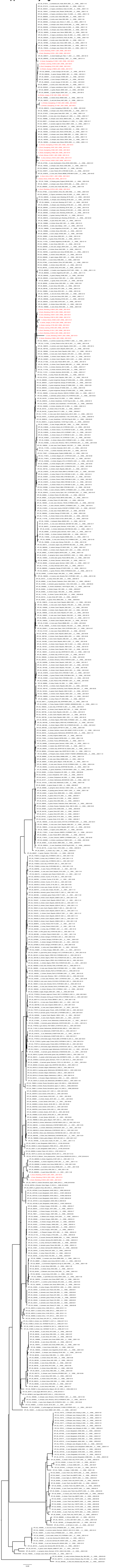

Supplementary Figure 6

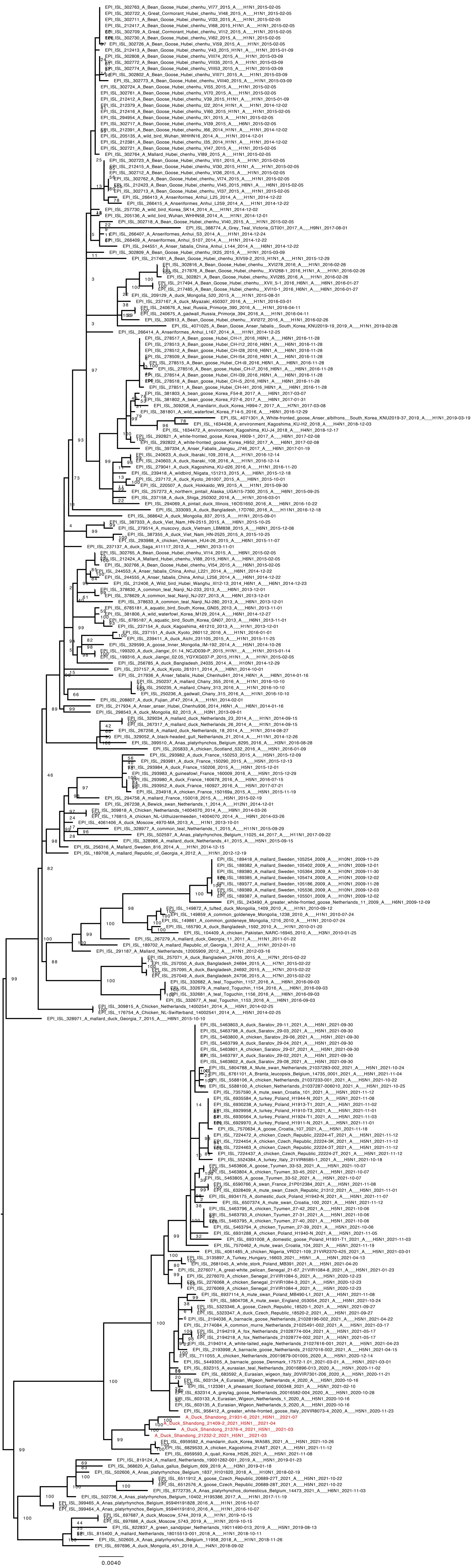

Supplementary Figure 7

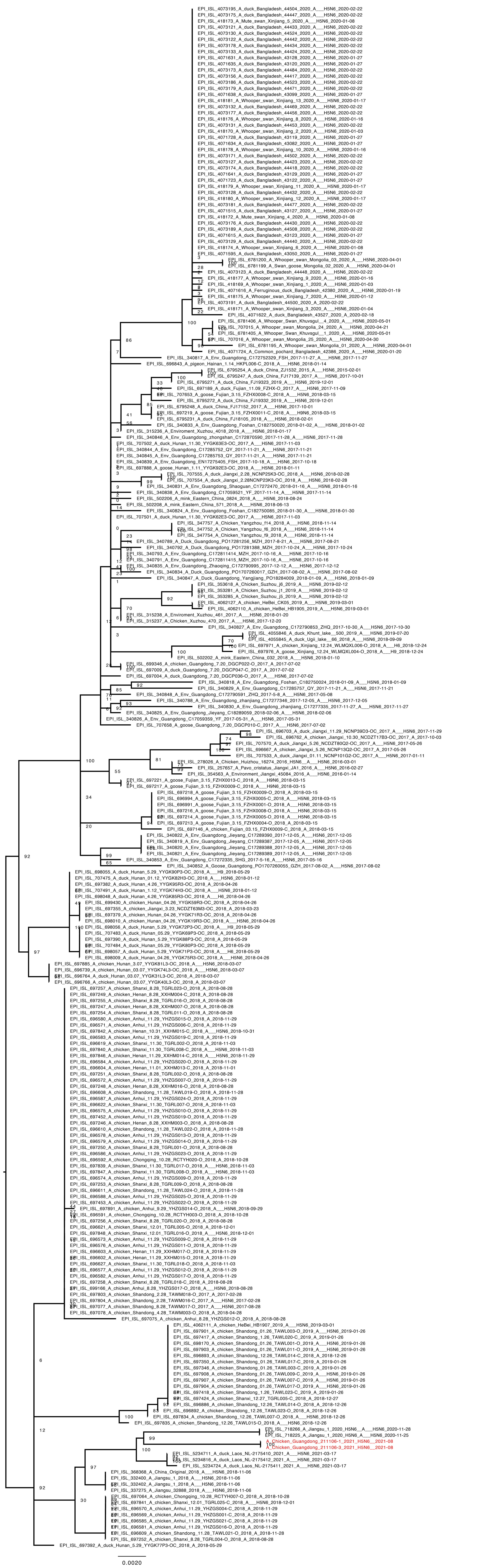

Supplementary Figure 8

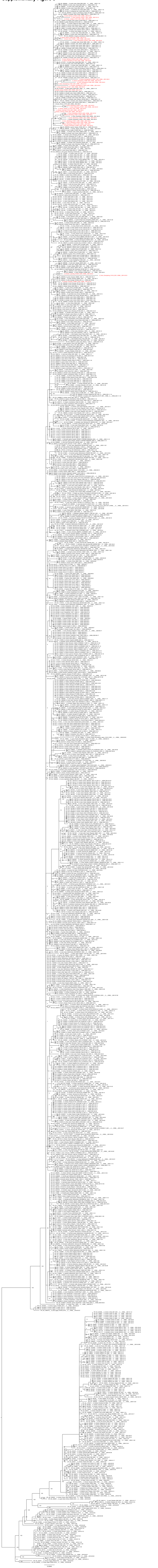

Supplementary Figure 9

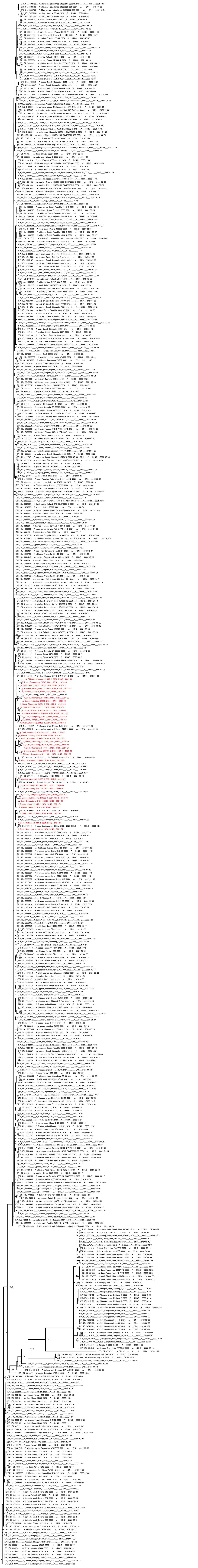

Supplementary Figure 10

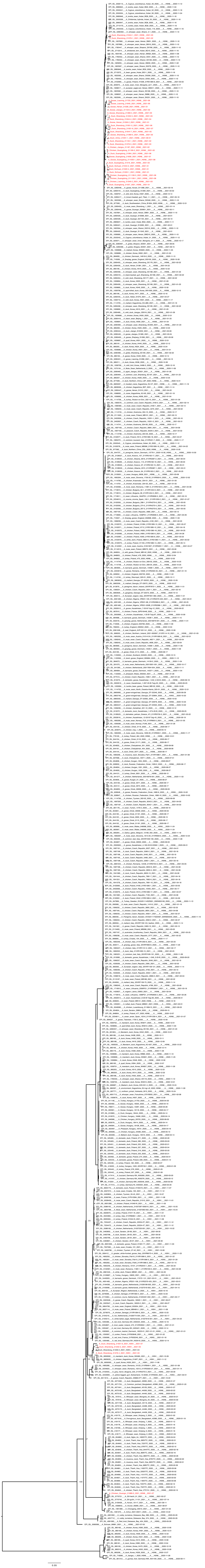

Supplement: Supplementary Figure 1 — Phylogenic tree of H5 subtype influenza viruses using PB2 gene sequences with available the other influenza viruses. The PB2 genes of H5 subtype viruses collected during 2021 in China together with the other influenza viruses were analyzed. The isolates with red color denote the H5N1, H5N6, and H5N8 viruses used in this study. The scale bar represents the number of nucleotide substitutions per site (subs/site). [file Data_Sheet_1.pdf]
